# Silylboronate-Mediated Defluorosilylation of Aryl Fluorides with or without Ni-Catalyst

**DOI:** 10.3389/fchem.2021.771473

**Published:** 2021-10-25

**Authors:** Jun Zhou, Zhengyu Zhao, Norio Shibata

**Affiliations:** ^1^ Department of Nanopharmaceutical Sciences, Nagoya Institute of Technology, Nagoya, Japan; ^2^ Institute of Advanced Fluorine-Containing Materials, Zhejiang Normal University, Jinhua, China

**Keywords:** defluorosilylation, transition-metal-free catalysis, C–F bond activation, silylboronate, nickel

## Abstract

The defluorosilylation of aryl fluorides to access aryl silanes was achieved under transition-metal-free conditions *via* an inert C–F bond activation. The defluorosilylation, mediated by silylboronates and KOtBu, proceeded smoothly at room temperature to afford various aryl silanes in good yields. Although a comparative experiment indicated that Ni catalyst facilitated this transformation more efficiently, the transition-metal-free protocol is advantageous from a green chemistry perspective.

## Introduction

Organofluorine compounds have been critical over the past few decades in pharmaceutical (Inoue et al., 2020), agrochemical (Ogawa et al., 2020), functional materials (Hiyama, 2000; Babudri et al., 2007; Berger et al., 2011; Liu et al., 2017; Liu et al., 2019) and polymer (Améduri et al., 2020) industries. The progress of synthetic technologies exemplified by fluorination (Rozen et al., 1996; Shibata et al., 2007; Furuya et al., 2011; Campbell et al., 2015; Ni et al., 2015; Lee et al., 2016; Zhu et al., 2018) and trifluoromethylation (Ma et al., 2004; Shibata et al., 2008; Shibata et al., 2010; Merino et al., 2014; Liu et al., 2015; Charpentier et al., 2015; Alonso et al., 2015; Xiao et al., 2021) reactions has expressively supported such success and prosperity of organofluorine compounds. One of the most attractive properties of organofluorine compounds is their durability, represented by Teflon^®^, induced by the most vital bond energy of the C–F bond in carbon chemistry (Uneyama et al., 2006; Luo et al., 2007; Amii et al., 2009). However, their robustness has often caused severe persistent environmental toxicity, such as the super-greenhouse effect by fluorocarbons (McCulloch et al., 2003; Velders et al., 2007; Shine and Sturges, 2007; Sovacool et al., 2021) and the bioaccumulation of perfluorooctanoic acid (PFOA) and perfluorooctanesulfonic acid (PFOS) (Vierke et al., 2012; Stanifer et al., 2018; Chen et al., 2019; Li et al., 2020). Given this limitation, recent attention has been focused on the activation and cleavage of remarkably inert C–F bonds of organofluorine molecules, creating a new field of research in fluorine chemistry (Stahl et al., 2013; Ahrens et al., 2015; Shen et al., 2015; Eisenstein et al., 2017; Hamel et al., 2018).

**SCHEME 1:**
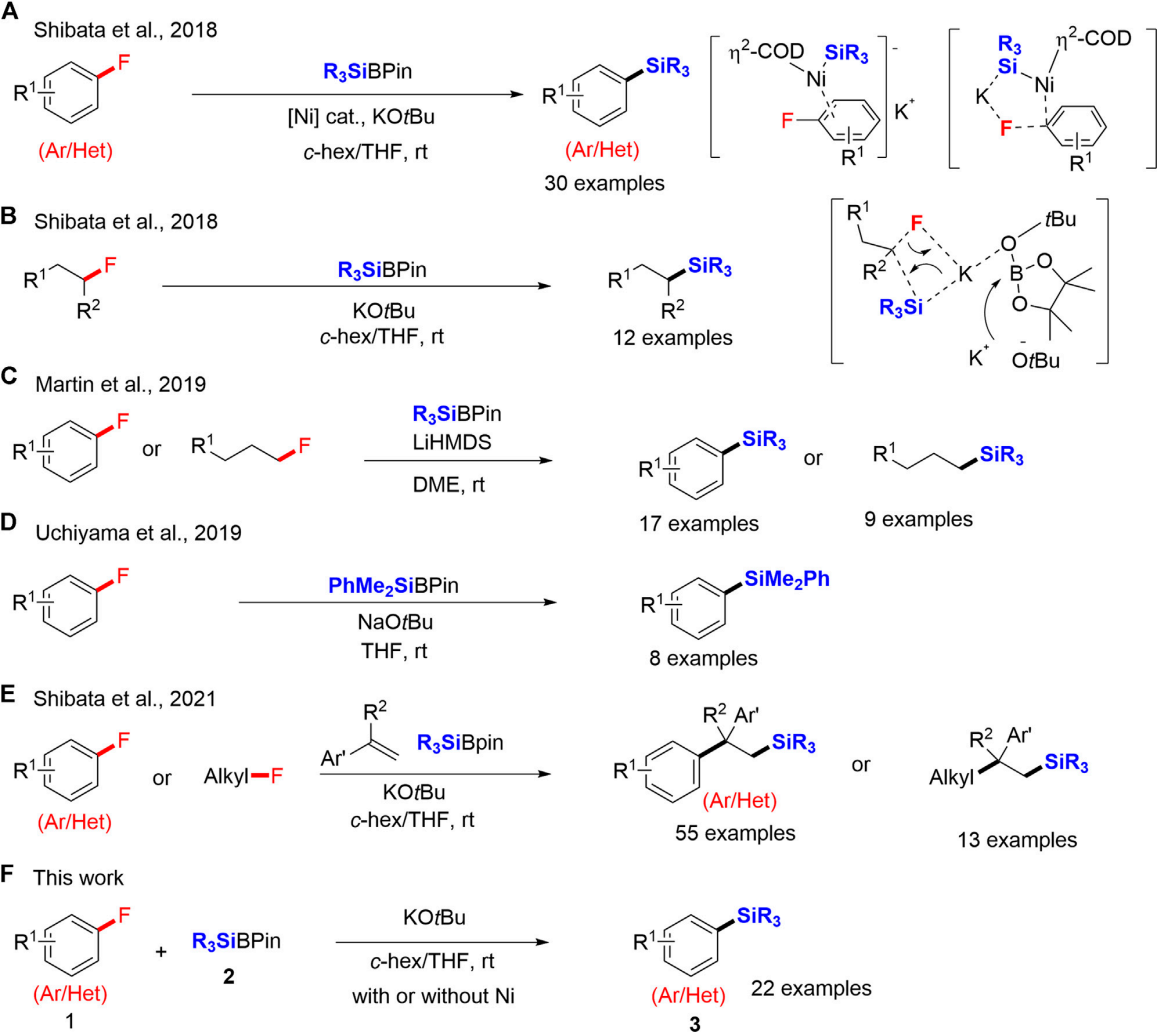
Examples of defluorosilylation reactions of organic fluorides with R_3_SiBPin.

In 2018, we reported a significant achievement on the C–F bond cleavage of aryl fluorides *via* defluorosilylation using silylboronates (R_3_SiBPin) in the presence of potassium *tert*-butoxide (KO*t*Bu) and a catalytic amount of Ni. The C–F bond cleavage occurred *via* the five-centered transition state *via* a *π*-nickel complex and a non-classical oxidative pathway (Scheme 1A); (Cui et al., 2018). Notably, we also found that the C–F bond activation did not require an Ni catalyst in the case of alkyl fluorides. The defluorosilylation of alkyl fluorides proceeded smoothly with R_3_SiBPin exclusively in the presence of KO*t*Bu. A highly nucleophilic, silyl anionic species directly reacts with alkyl fluorides *via* a concerted S_N_2 process (Scheme 1B). The defluorosilylation reaction was then successfully reported by several groups (Gao et al., 2019; Liu et al., 2019; Kojima et al., 2019; Mallick et al., 2019; Coates et al., 2019; Lim et al., 2020; Sheldon et al., 2020). In 2019, Martin and co-workers reported the lithium-promoted defluorosilylation of organic fluorides, in which lithium bis(trimethylsilyl)amide (LiHMDS) and dimethyl ether (DME) cooperated well to activate the inert C–F bond (Scheme 1C); (Liu et al., 2019). In the same year, Uchiyama and co-workers also reported a transition-metal-free defluorosilylation of fluoroarenes using PhMe_2_SiBPin and sodium *tert*-butoxide (NaO*t*Bu) (Kojima et al., 2019). *In situ* generated silyl anion species enabled the direct defluorosilylation of fluoroarenes (Scheme 1D). In 2021, we have continuously reported the catalyst-free carbosilylation of alkenes using R_3_SiBPin and organic fluorides, including aryl and alkyl fluorides, *via* selective C–F bond activation (Zhou et al., 2021). The substrate-scope showed slightly better yields when the reaction was performed in the presence of an Ni-catalyst, although we noticed that the effect of Ni-catalyst was not significant (Scheme 1E). While the results of Uchiyama and co-workers (Scheme 1D); (Kojima et al., 2019) and our recent results (Scheme 1E); (Zhou et al., 2021) indicate that Ni-catalyst is not necessary for their transformations, the conditions are not precisely the same such as bases, solvents and reaction times, which is difficult to conclude the Ni-effect. We thus decided to carefully re-examine our original work of defluorosilylation of aryl fluorides in 2018 (Scheme 1A); (Cui et al., 2018) by the same conditions, R_3_SiBPin in the presence of KO*t*Bu, with or without an Ni-catalyst. We disclose herein the improved-catalyst-free conditions for silylboronate-mediated defluorosilylation of aryl fluorides. A wide variety of aryl fluorides **1** having a substitution at the aromatic ring were smoothly converted into the corresponding aryl silanes **3** in good yields by R_3_SiBPin **2** (2.0 equiv) in the presence of KO*t*Bu (3.0 equiv) in a mixed solvent system (*c*-hex/THF = 1/2) at room temperature. Heteroaromatic fluorides **1** are also accepted by the same conditions to provide heteroaromatic silanes **3** in good yields. We also carried out the same reactions under Ni-catalysis. While the yields under the catalyst-free conditions were lower than those under Ni-catalysis, the transition-metal-free system is advantageous from the perspective of green chemistry (Scheme 1F).

## Results and Discussion

To start the optimization, we selected 4-fluorobiphenyl (**1a**) and silylboronate Et_3_SiBpin (**2a**) as model substrates to examine the defluorosilylation reaction. Based on our earlier reported conditions of the Ni-catalyzed defluorinative silylation of aryl fluorides **1** [Et_3_SiBpin (1.5 equiv), KO*t*Bu (2.5 equiv), 10 mol% Ni(cod)_2_ in cyclohexane (*c*-hex)/THF (1/2, *v/v*) at room temperature], we carried out the reaction of **1a** with **2a** under the conditions mentioned above but without Ni-catalyst. All the optimizations were carried out on a 0.1 mmol scale of **1a**. The expected biphenyl-4-yl-triethylsilane (**3aa**) was observed in 65% ^1^H NMR yield after 8 h (entry 1, Table 1). To compare Uchiyama’s reaction conditions (Kojima et al., 2019) (NaO*t*Bu, THF), replacing KO*t*Bu with NaO*t*Bu, gave 58% yield of **3aa** (entry 2). Other bases such as LiO*t*Bu or KOMe resulted in no reaction (entries 3 and 4). The conditions by Martin (Liu et al., 2019) (LiHMDS, DME) were also attempted but using our solvent system (*c*-hex/THF = 1/2, *v/v*), but no reaction resulted (entry 5). Interestingly, KHMDS facilitated this defluorosilylation reaction by affording **3aa** in 27% yield (entry 6). We subsequently attempted the reaction in a single solvent of *c*-hex, THF, or diglyme to investigate the effect of solvent. The mixed solvent system, *c*-hex/THF (entry 1), was more effective than others (entries 7–9). We next varied the amounts of **2a** and KO*t*Bu (entries 10 and 11) and found that 2.0 equiv of **2a** and 3.0 equiv of KO*t*Bu were the optimum amounts to afford **3aa** in 74% yield (56% isolated yield; entry 11). To re-ascertain the effect of Ni(COD)_2_, we investigated the reaction using these optimized conditions (entry 11) but in the presence of Ni catalyst. The defluorosilylation reaction performed more efficiently under the optimal conditions with Ni(COD)_2_ to give **3aa** in 83% yield (65% isolated yield; entry 12), while **1a** remained (detected by crude ^19^F NMR). These comparative results thus convinced us that Ni(COD)_2_ accelerates the present defluorinative transformation, while the transition-metal-free variant (entry 11) is advantageous from a green chemistry perspective.

**TABLE 1:** Optimization of defluorosilylation reaction conditions.

**Table:** 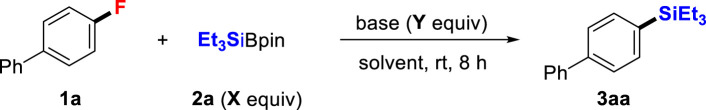

Entry	X	Base (Y)	Solvent	Yield of 3aa^a^
1	1.5	KO*t*Bu (2.5)	*c*-hex/THF (1/2)	65%
2	1.5	NaO*t*Bu (2.5)	*c*-hex/THF (1/2)	58%
3	1.5	LiO*t*Bu (2.5)	*c*-hex/THF (1/2)	N.R.
4	1.5	KOMe (2.5)	*c*-hex/THF (1/2)	N.R.
5	1.5	LiHMDS (2.5)	*c*-hex/THF (1/2)	N.R.
6	1.5	KHMDS (2.5)	*c*-hex/THF (1/2)	27%
7	1.5	KO*t*Bu (2.5)	*c*-hex	45%
8	1.5	KO*t*Bu (2.5)	THF	62%
9	1.5	KO*t*Bu (2.5)	diglyme	45%
10	1.5	KO*t*Bu (3.0)	*c*-hex/THF (1/2)	60%
11	2.0	KO*t*Bu (3.0)	*c*-hex/THF (1/2)	74% (56%)^c^
12^b^	2.0	KO*t*Bu (3.0)	*c*-hex/THF (1/2)	83% (65%)^c^

^a^Unless otherwise noted, the reaction was carried out using **1a** (0.1 mmol), Et_3_SiBpin (**2a**), and a base in solvent (0.6 ml, *v/v*) at rt for 8 h; yields were determined by ^1^H NMR and ^19^F NMR analysis of the crude reaction mixture using 3-fluoropyridine as the internal standard.

b 10 mol% Ni(cod)_2_ was added.

c Isolated yield is shown in parentheses.

With the optimized reaction conditions in hand (entry 11, Table 1), we next examined the feasibility of this transition-metal-free defluorosilylation reaction (Table 2). All the reactions were carried out on a 0.2 mmol scale of **1**. As shown, various aromatic fluorides were examined under catalyst-free conditions. We efficiently converted a wide range of fluoroarenes **1** into corresponding defluorosilylation products **3** in good yield. It was found that any position (*o-*, *m*-, or *p*-) in the aromatic substitution of **1** was viable, affording the corresponding products **3** (**3aa**: 59%; **3ba**: 51%; **3ca**: 26%; **3da**: 40%; **3ea**: 55%) in acceptable to good yields (26–59%) under the catalyst-free conditions. We next repeated the same substrate scope in the presence of Ni(COD)_2_ (entry 12, Table 1) and the yield of products **3aa**–**3ea** improved considerably (**3aa**: 86%; **3ba**: 82%; **3ca**: 74%; **3da**: 70%; **3ea**: 79%). Thus, these differences clearly show the efficiency of Ni(COD)_2_. Previous results with Ni(COD)_2_ are also indicated in Table 1 to ascertain the advantage of the Ni catalyst. Besides, the aryl fluorides **1f**–**1h** with an electron-rich substitution were well-tolerated in this defluorosilylation reaction in moderate yield (**3fa**: 46%; **3ga**: 45%; **3ha**: 39%). Several substituted aryl silanes (**3ia**–**3na**) were also successfully obtained in moderate yield under identical conditions and a variety of functional groups such as OMe (**1j**), OMOM (**1k**), OPh (**1l**), NMe_2_ (**1m**) and 1*H*-pyrrole (**1n**) were well tolerated. The nitrogen-containing hetero-aromatic fluorides **1o**–**1q** were successfully converted to the corresponding silanes **3**. For example, 5-fluoro-2-phenylpyridine (**1o**) and 1*H*-indole derivatives (**1p** and **1q**), which possess an active C–H bond, were well-tolerated and smoothly underwent the selective defluorosilylation process to afford desired products (**3oa**: 43%; **3pa**: 37%; **3qa**: 42%). Notably, 1,2-difluorobenzene (**1r**) was efficiently mono-silylated in good yield (**3ra**: 62%). Sterically demanding *o*-substituted substrates **1s** and **1t** were also transformed into the corresponding products **3sa** and **3ta** under Ni-free conditions in 26 and 12% yields, respectively. Ni-catalyst conditions improved both cases to 67% (**3sa**) and 35% (**3ta**). Furthermore, other silyl boronates such as PhMe_2_SiBpin (**2b**) and *t*BuMe_2_SiBPin (**2c**) were also investigated instead of **2a** to yield the corresponding silylated products **3ab** and **3ac** in 36 and 51% yield, respectively. In all cases, the Ni catalyst-based protocol (Cui et al., 2018) has a substantial yield advantage in this defluorosilylation reaction, while both conditions did not entirely consume the staring materials **1**. The substrates (**1u** and **1v**) having electron-withdrawing group were not suitable, which is the limitation of this transformation.

**TABLE 2:** Substrate scope of the defluorosilylation strategies^a^.

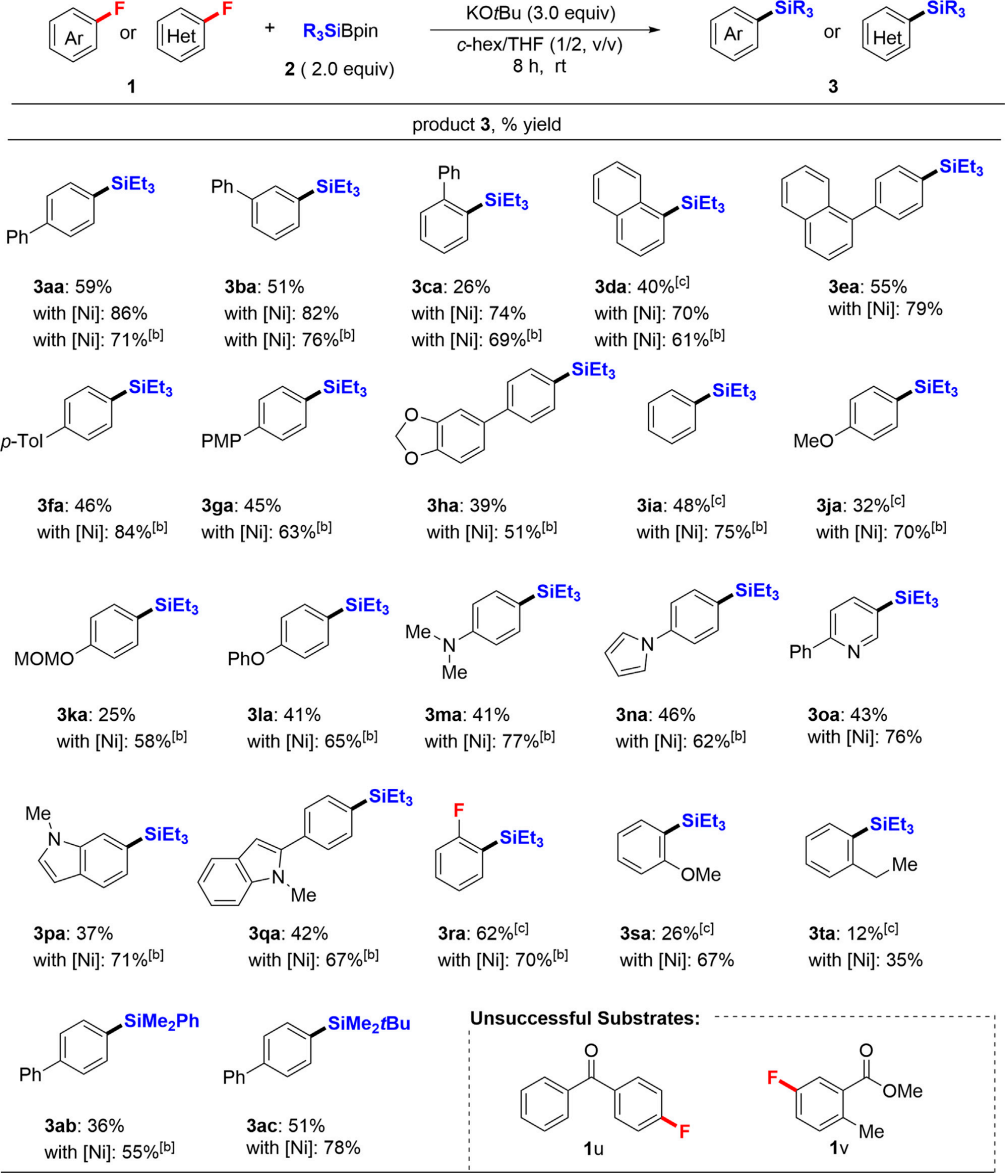

a Unless otherwise noted, the reaction was carried using **1** (0.2 mmol), **2** (2.0 equiv), and KO*t*Bu (3.0 equiv) without or with Ni(COD)_2_ (10 mol%) in *c*-hex/THF (1.2 ml, 1/2, *v/v*) at rt for 8 h. Isolated yields are shown.

b The yields shown are previously reported data by using reaction conditions: **1** (0.2 mmol), **2** (1.5 equiv), Ni(COD)_2_ (10 mol%), KO*t*Bu (2.5 equiv), *c*-hex/THF (0.8 ml, 1/2, *v/v*), rt, 2–12 h.

c 0.4 mmol **1** was used.

PMP, *p*-methoxyphenyl; MOM, methoxymethyl.

**SCHEME 2:**
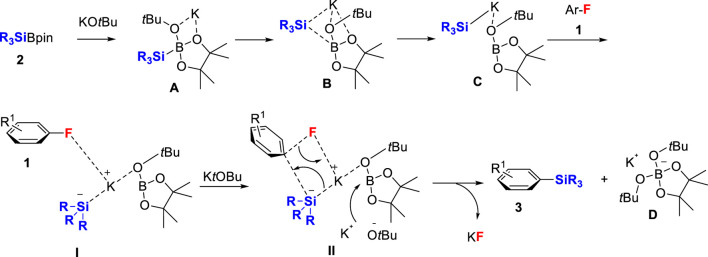
A schematic of the reaction process of catalyst-free defluorosilylation of aryl fluorides **1** with R_3_SiBPin **2** in the presence of *t*BuOK.

Based on our previous work of defluorosilylation of alkyl fluorides **1** with R_3_SiBPin **2** mediated by a potassium base (Cui et al., 2018), the defluorosilylation of aryl fluorides mediated by a lithium base (Martin) (Liu et al., 2019) and by a sodium base (Uchiyama) (Kojima et al., 2019), the reaction should proceed the nucleophilic attack of the silyl anion involving a concerted S_N_Ar process. A schematic reaction of the catalyst-free defluorosilylation process is presented in Scheme 2 by considering our previous work and Uchiyama’s elegant DFT calculations (Kojima et al., 2019). First, R_3_SiBPin **2** reacts with *t*BuOK to provide potassium silyl anion species **C** complexed with *t*BuO-BPin *via* **A** and **B** (Cui et al., 2018; Jain et al., 2018; Zhou et al., 2021). **C** approaches the aryl fluoride **1** to form the intermediate **I**. A concerted S_N_Ar reaction happens with the attack of the boron center of *t*BuO-BPin by another *t*BuOK *via* a transition state **II** with the key C–F bond cleavage to furnish the aryl silanes **3** with the formation of KF and **D**, K^+^[*t*BuO_2_BPin]-.

## Conclusion

In summary, we reported a feasible transition-metal-free method for synthesizing aryl silanes **3** through the defluorosilylation of aryl fluorides **1** by using silylboronates R_3_SiBPin **2** and KO*t*Bu. Furthermore, we compared our new results with a previous report on the success of Ni-catalyzed defluorosilylation of fluoroarenes. Thus, we concluded that the transformation of aryl fluorides into corresponding aryl silanes *via* a C**−**F bond cleavage can be achieved even in the absence of Ni(COD)_2_, but in relatively lower yields than those of the Ni-catalyzed protocol, due to different reaction mechanisms. A further extension of this methodology is currently underway.

## Data Availability

The original contributions presented in the study are included in the article/Supplementary Material, further inquiries can be directed to the corresponding author.
