# Supplementary material for: Silylboronate-Mediated Defluorosilylation of Aryl Fluorides with or without Ni-Catalyst
Source: Front Chem. 2021 Oct 25;9:771473. doi: 10.3389/fchem.2021.771473 (PMC8573161; doi:10.3389/fchem.2021.771473)

Supplementary Material

1. General Information

All reactions were performed in oven-dried glassware under a positive pressure of nitrogen. Solvents were transferred via syringe and were introduced into the reaction vessels through a rubber septum. Column chromatography was carried out on a column packed with silica-gel 60N spherical neutral size 63-210. The ^{1}H NMR (300, 500 MHz), ^{19}F NMR (282 MHz), ^{13}C NMR (126 MHz) spectra for the solution in $CDCl_3$ were recorded on a Varian Mercury 300, Bruker Avance 500 NMR spectrometers. Chemical shifts (δ) are expressed in ppm downfield from internal TMS (δ = 0.00 ppm), $CHCl_3$ (δ = 7.26 ppm for ^{1}H NMR, δ = 77.16 ppm for ^{13}C NMR), C_6F_6 (δ = -162.2 ppm for ^{19}F NMR). Mass spectra were recorded on a SHIMADZU GCMS-QP5050A (EI-MS). Infrared spectra were recorded on a JASCO FT/IR-4100 spectrometer.

Commercially available chemicals were obtained from Aldrich Chemical Co., Alfa Aesar, TCI and used as received unless otherwise noted. Solvents such as ethyl acetate, Dioxane, Diglyme, Et_2O and THF were dried and distilled before use.

Fluoroarenes **1a**, **1c**, **1d**, **1i**, **1j**, **1r**, and **1s** were purchased from TCI or Sigma Aldrich. **1b**, **1e**, **1f**, **1g**, **1h**, **1k**, **1l**, **1m**, **1n**, **1o**, **1p**, **1q**, and **1t** were used prepared according to known methods.[1 and 2] Silylboronates **2a**, **2b**, and **2c** were used prepared according to previous methods.[2]

2. General procedure for the optimization of defluorosilylation reactions

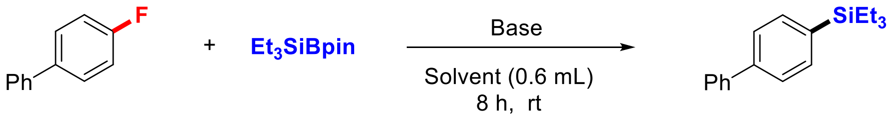

1a **2a** **3aa**

In a N_2 filled glovebox, to a flame-dried screw-capped test tube were added Aryl fluorides **1a** (0.10 mmol, 1.0 equiv), with or without $Ni(cod)_2$ (10 mol %), indicated amount of Silyl boronates **2a**, base and solvent (0.6 mL) sequentially. The tube then was sealed and removed from the glovebox. The solution was stirred at room temperature for 8h. The reaction tube was added *n*-Hexane (5 mL), then subject to filter through a short silica pad, and washed with Et_2O, concentrated under vacuum, followed by 3-Fluoropyridine (8.6 μL, 0.1 mmol) as an internal standard. After NMR analysis was conducted. The mixture was then concentrated again to give the residue, which was purified by column chromatography on silica gel to give the corresponding arylsilanes **3aa**.

3. General procedure for the defluorosilylation of aryl fluorides

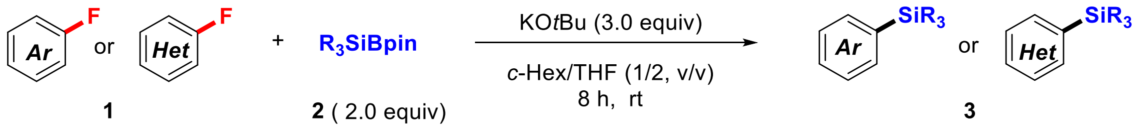

In a N_2 filled glovebox, to a flame-dried screw-capped test tube were added Aryl fluorides **1** (0.20 mmol, 1.0 equiv), Silyl boronates **2** (0.4 mmol, 2.0 equiv), with or without $Ni(cod)_2$ (10 mol %), KO*t*Bu (67 mg, 0.6 mmol, 3.0 equiv) and cyclohexane/THF (1.2 mL, 1/2, *v/v*) sequentially. The tube then was sealed and removed from the glovebox. The solution was stirred at room temperature for 8h. The reaction tube was added *n*-Hexane (5 mL), then subject to filter through a short silica pad, and washed with Et_2O, concentrated under vacuum, followed by 3-Fluoropyridine (8.6 μL, 0.1 mmol) as an internal standard. After NMR analysis was conducted. The mixture was then concentrated again to give the residue, which was purified by column chromatography on silica gel to give the corresponding arylsilanes **3**.

Biphenyl-4-yltriethylsilane (3aa)[1]

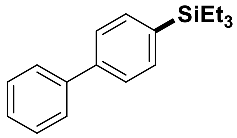

Compound **3aa** was obtained as a colorless oil (without Nickel catalysis: 31.8 mg, Yield: 59%; with Nickel catalysis: 46.1 mg, Yield: 86%). **^{1}H NMR** (300 MHz, $CDCl_3$) δ 7.72 – 7.55 (m, 5H), 7.51 – 7.41 (m, 3H), 7.39 – 7.32 (m, 1H), 1.01 (t, *J* = 7.7 Hz, 9H), 0.83 (q, *J* = 8.9, 8.3 Hz, 6H). **MS** (EI) *m*/*z* $[M]^+$: 268. The chemical shifts were consistent with those reported in the literature.

Biphenyl-3-yltriethylsilane (3ba)[1]

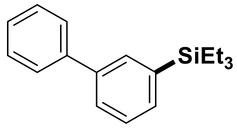

Compound **3ba** was obtained as a colorless oil (without Nickel catalysis: 27.4 mg, Yield: 51%; with Nickel catalysis: 44 mg, Yield: 82%). **^{1}H NMR** (300 MHz, $CDCl_3$) δ 7.72 (s, 1H), 7.64 – 7.56 (m, 3H), 7.54 – 7.42 (m, 4H), 7.38 (d, *J* = 7.1 Hz, 1H), 1.02 (t, *J* = 7.5 Hz, 9H), 0.92 – 0.82 (m, 6H). **MS** (EI) *m*/*z* $[M]^+$: 268. The chemical shifts were consistent with those reported in the literature.

Biphenyl-2-yltriethylsilane (3ca)[1]

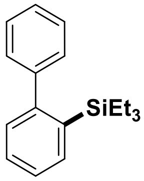

Compound **3ca** was obtained as a colorless oil (without Nickel catalysis: 14 mg, Yield: 26%; with Nickel catalysis: 40 mg, Yield: 74%). **^{1}H NMR** (300 MHz, $CDCl_3$) δ 7.70 (s, 1H), 7.64 – 7.55 (m, 3H), 7.46 (t, *J* = 7.4 Hz, 3H), 7.39 – 7.33 (m, 2H), 1.01 (t, *J* = 7.6 Hz, 9H), 0.92 – 0.80 (m, 6H). **MS** (EI) *m*/*z* $[M]^+$: 268. The chemical shifts were consistent with those reported in the literature.

Triethyl(naphthalen-1-yl)silane (3da)[1]

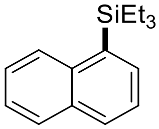

Compound **3da** was obtained as a colorless oil (without Nickel catalysis: 38.7 mg, Yield: 40%; with Nickel catalysis: 67.8 mg, Yield: 70%). **^{1}H NMR** (300 MHz, $CDCl_3$) δ 8.20 – 8.06 (m, 1H), 7.96 – 7.79 (m, 2H), 7.69 (dt, *J* = 6.8, 1.2 Hz, 1H), 7.55 – 7.42 (m, 3H), 1.13 – 0.89 (m, 15H). **MS** (EI) *m*/*z* $[M]^+$: 242. The chemical shifts were consistent with those reported in the literature.

Triethyl(4-(naphthalen-1-yl)phenyl)silane (3ea)[3]

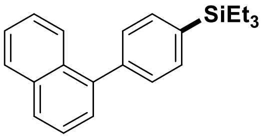

Compound **3ea** was obtained as a colorless oil (without Nickel catalysis: 35.1 mg, Yield: 55%; with Nickel catalysis: 50 mg, Yield: 79%). **^{1}H NMR** (300 MHz, $CDCl_3$) δ 7.90 – 7.83 (m, 2H), 7.67 (dd, *J* = 8.4, 1.2 Hz, 1H), 7.51 – 7.43 (m, 3H), 7.35 (dt, *J* = 4.5, 1.1 Hz, 1H), 7.33 – 7.24 (m, 2H), 7.22 – 7.09 (m, 2H), 0.93 – 0.73 (m, 9H), 0.50 (q, *J* = 7.8 Hz, 6H). **MS** (EI) *m*/*z* $[M]^+$: 318. The chemical shifts were consistent with those reported in the literature.

Triethyl(4'-methylbiphenyl-4-yl)silane (3fa)[1]

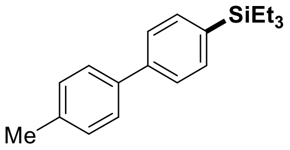

Compound **3fa** was obtained as a white solid (26 mg, Yield: 46%). **^{1}H NMR** (300 MHz, $CDCl_3$) δ 7.67 – 7.46 (m, 6H), 7.26 (d, *J* = 7.9 Hz, 2H), 2.40 (s, 3H), 1.00 (t, *J* = 7.7 Hz, 9H), 0.90 – 0.66 (m, 6H). **MS** (EI) *m*/*z* $[M]^+$: 282. The chemical shifts were consistent with those reported in the literature.

Triethyl(4'-methoxybiphenyl]-4-yl)silane (3ga)[1]

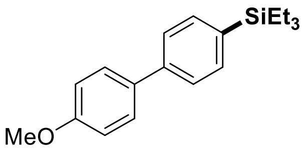

Compound **3ga** was obtained as a white solid (26.8 mg, Yield: 45%). **^{1}H NMR** (300 MHz, $CDCl_3$) δ 7.66 – 7.40 (m, 6H), 6.99 (d, *J* = 8.8 Hz, 2H), 3.86 (s, 3H), 1.01 (t, *J* = 7.8 Hz, 9H), 0.93 – 0.68 (m, 6H). **MS** (EI) *m*/*z* $[M]^+$: 298. The chemical shifts were consistent with those reported in the literature.

(4-(Benzo[*d*][1,3]dioxol-5-yl)phenyl)triethylsilane (3ha)[1]

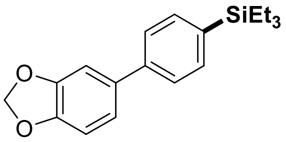

Compound **3ha** was obtained as a white solid (24.4 mg, Yield: 39%). **^{1}H NMR** (300 MHz, $CDCl_3$) δ 7.63 – 7.37 (m, 4H), 7.10 – 7.03 (m, 2H), 6.94 – 6.83 (m, 1H), 6.00 (s, 2H), 0.99 (t, *J* = 7.7 Hz, 9H), 0.81 (q, *J* = 7.7 Hz, 6H). **MS** (EI) *m*/*z* $[M]^+$: 312. The chemical shifts were consistent with those reported in the literature.

Triethyl(phenyl)silane (3ia)[1]

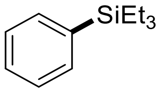

Compound **3ia** was obtained as a colorless oil (36.5 mg, Yield: 48%). **^{1}H NMR** (300 MHz, $CDCl_3$) δ 7.55 – 7.47 (m, 2H), 7.37 (dd, *J* = 3.9, 2.4 Hz, 3H), 0.99 (t, *J* = 7.7 Hz, 9H), 0.82 (q, *J* = 7.3, 6.8 Hz, 6H). **MS** (EI) *m*/*z* $[M]^+$: 192. The chemical shifts were consistent with those reported in the literature.

Triethyl(4-methoxyphenyl)silane (3ja)[1]

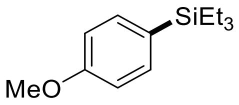

Compound **3ja** was obtained as a colorless oil (28 mg, Yield: 32%). **^{1}H NMR** (300 MHz, $CDCl_3$) δ 7.56 – 7.21 (m, 2H), 7.12 – 6.83 (m, 2H), 3.82 (s, 3H), 1.04 – 0.89 (m, 9H), 0.85 – 0.71 (m, 6H). **MS** (EI) *m/z* $[M]^+$: 222. The chemical shifts were consistent with those reported in the literature.

Triethyl(4-(methoxymethoxy)phenyl)silane (3ka)[1]

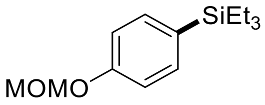

Compound **3ka** was obtained as a colorless oil (12.6 mg, Yield: 25%). **^{1}H NMR** (300 MHz, $CDCl_3$) δ 7.41 (d, *J* = 8.7 Hz, 2H), 7.03 (d, *J* = 8.6 Hz, 2H), 5.19 (s, 2H), 3.48 (s, 3H), 1.05 – 0.86 (m, 9H), 0.82 – 0.67 (m, 6H). **MS** (EI) *m/z* $[M]^+$: 252. The chemical shifts were consistent with those reported in the literature.

Triethyl(4-phenoxyphenyl)silane (3la)[1]

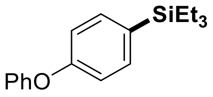

Compound **3la** was obtained as a colorless oil (23.3 mg, Yield: 41%). **^{1}H NMR** (300 MHz, $CDCl_3$) δ 7.44 (d, *J* = 7.8 Hz, 2H), 7.33 (q, *J* = 6.4, 5.2 Hz, 2H), 7.11 (t, *J* = 7.9 Hz, 1H), 7.06 – 6.94 (m, 4H), 0.96 (t, *J* = 7.7 Hz, 9H), 0.78 (q, *J* = 8.7, 7.8 Hz, 6H). **MS** (EI) *m/z* $[M]^+$: 284. The chemical shifts were consistent with those reported in the literature.

***N*,*N*-Dimethyl-4-(triethylsilyl)aniline (3ma)**[1]

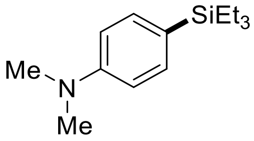

Compound **3ma** was obtained as a colorless oil (19.3 mg, Yield: 41%). **^{1}H NMR** (300 MHz, $CDCl_3$) δ 7.30 – 7.17 (m, 1H), 6.92 – 6.82 (m, 2H), 6.82 – 6.69 (m, 1H), 2.96 (s, 6H), 0.99 (t, *J* = 7.7 Hz, 9H), 0.80 (q, *J* = 7.5 Hz, 6H). **MS** (EI) *m/z* $[M]^+$: 235. The chemical shifts were consistent with those reported in the literature.

1-(4-(Triethylsilyl)phenyl)-1*H*-pyrrole (3na)[1]

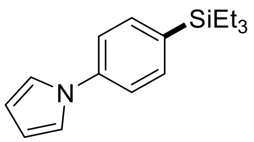

Compound **3na** was obtained as a colorless oil (23.8 mg, Yield: 46%). **^{1}H NMR** (300 MHz, $CDCl_3$) δ 7.54 (d, *J* = 8.5 Hz, 2H), 7.38 (d, *J* = 8.4 Hz, 2H), 7.12 (t, *J* = 2.2 Hz, 2H), 6.35 (t, *J* = 2.2 Hz, 2H), 1.03 – 0.92 (m, 9H), 0.88 – 0.76 (m, 6H). **MS** (EI) *m*/*z* $[M]^+$: 257. The chemical shifts were consistent with those reported in the literature.

2-Phenyl-5-(triethylsilyl)pyridine (3oa)[4]

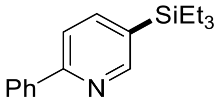

Compound **3oa** was obtained as a colorless oil (22.8 mg, Yield: 43%). **^{1}H NMR** (300 MHz, $CDCl_3$) δ 8.43 (s, 1H), 7.91 (dd, *J* = 6.8, 1.6 Hz, 2H), 7.68 (dd, *J* = 4.1, 1.5 Hz, 1H), 7.56 – 7.33 (m, 4H), 1.10 – 0.78 (m, 15H). **MS** (EI) *m*/*z* $[M]^+$: 269. The chemical shifts were consistent with those reported in the literature.

1-Methyl-6-(triethylsilyl)-1*H*-indole (3pa)[1]

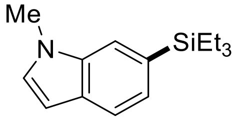

Compound **3pa** was obtained as a colorless oil (18.2 mg, Yield: 37%). **^{1}H NMR** (300 MHz, $CDCl_3$) δ 7.63 (d, *J* = 7.8 Hz, 1H), 7.44 (s, 1H), 7.22 (d, *J* = 7.9 Hz, 1H), 7.05 (d, *J* = 3.1 Hz, 1H), 6.47 (d, *J* = 3.2 Hz, 1H), 3.82 (s, 3H), 1.00 (t, *J* = 7.6 Hz, 9H), 0.90 – 0.79 (m, 6H). **MS** (EI) *m*/*z* $[M]^+$: 245. The chemical shifts were consistent with those reported in the literature.

1-Methyl-2-(4-(triethylsilyl)phenyl)-1*H*-indole (3qa)[1]

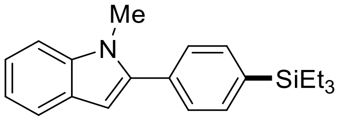

Compound **3qa** was obtained as a colorless oil (30 mg, Yield: 42%). **^{1}H NMR** (300 MHz, $CDCl_3$) δ 7.61 (dd, *J* = 15.4, 7.8 Hz, 3H), 7.49 (d, *J* = 7.7 Hz, 2H), 7.36 (d, *J* = 8.2 Hz, 1H), 7.24 (t, *J* = 7.7 Hz,

1H), 7.14 (t, J = 7.4 Hz, 1H), 6.57 (s, 1H), 3.76 (s, 3H), 1.01 (t, J = 7.7 Hz, 9H), 0.84 (q, J = 7.2 Hz, 6H). **MS** (EI) m/z $[M]^+$: 321. The chemical shifts were consistent with those reported in the literature.

Triethyl(2-fluorophenyl)silane (3ra)[1]

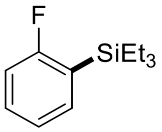

Compound **3ra** was obtained as a colorless oil (52.1 mg, Yield: 62%). ^{1}**H NMR** (300 MHz, $CDCl_3$) δ 7.45 – 7.28 (m, 2H), 7.13 (tt, J = 7.3, 1.0 Hz, 1H), 7.05 – 6.93 (m, 1H), 0.97 (t, J = 7.6 Hz, 9H), 0.90 – 0.80 (m, 6H). ^{19}**F NMR** (282 MHz, $CDCl_3$) δ -99.99 (q, J = 6.7 Hz). **MS** (EI) m/z $[M]^+$: 210. The chemical shifts were consistent with those reported in the literature.

Triethyl(2-methoxyphenyl)silane (3sa)[5]

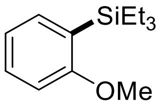

Compound **3sa** was obtained as a colorless oil (without Nickel catalysis: 23 mg, Yield: 26%; with Nickel catalysis: 59.1 mg, Yield: 67%). ^{1}**H NMR** (300 MHz, $CDCl_3$) δ 7.35 (t, J = 8.0 Hz, 2H), 6.95 (t, J = 7.2 Hz, 1H), 6.83 (d, J = 8.1 Hz, 1H), 3.79 (s, 3H), 0.95 (t, J = 7.5 Hz, 9H), 0.82 (q, J = 6.6 Hz, 6H). **MS** (EI) m/z $[M]^+$: 222. The chemical shifts were consistent with those reported in the literature.

Triethyl(2-ethylphenyl)silane (3ta)[6]

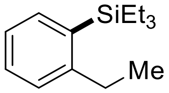

Compound **3ta** was obtained as a colorless oil (without Nickel catalysis: 10.5 mg, Yield: 12%; with Nickel catalysis: 30.5 mg, Yield: 35%). ^{1}**H NMR** (300 MHz, $CDCl_3$) δ 7.42 (d, J = 7.4 Hz, 1H), 7.32 (t, J = 7.4 Hz, 1H), 7.24 (t, J = 7.3 Hz, 1H), 7.16 (t, J = 7.3 Hz, 1H), 2.73 (q, J = 7.4 Hz, 2H), 1.24 (t, J = 7.5 Hz, 3H), 0.95 (t, J = 7.0 Hz, 16H), 0.85 (q, J = 7.8, 7.2 Hz, 12H). **MS** (EI) m/z $[M]^+$: 220. The chemical shifts were consistent with those reported in the literature.

Biphenyl-4-yldimethyl(phenyl)silane (3ab)[1]

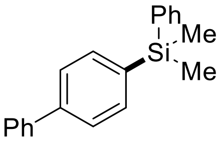

Compound **3ab** was obtained as a colorless oil (21 mg, Yield: 36%). **^{1}H NMR** (300 MHz, $CDCl_3$) δ 7.65 – 7.54 (m, 8H), 7.45 (t, *J* = 7.4 Hz, 2H), 7.42 – 7.34 (m, 4H), 0.60 (s, 6H). **MS** (EI) *m*/*z* $[M]^+$: 288. The chemical shifts were consistent with those reported in the literature.

Biphenyl-4-yl(*tert*-butyl)dimethylsilane (3ac)[7]

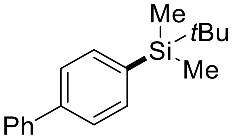

Compound **3ac** was obtained as a colorless oil (without Nickel catalysis: 27.5 mg, Yield: 51%; with Nickel catalysis: 41.8 mg, Yield: 78%). **^{1}H NMR** (300 MHz, $CDCl_3$) δ 7.66 – 7.57 (m, 5H), 7.45 (t, *J* = 7.3 Hz, 2H), 7.35 (t, *J* = 7.4 Hz, 2H), 0.92 (s, 9H), 0.31 (s, 6H). **MS** (EI) *m*/*z* $[M]^+$: 268. The chemical shifts were consistent with those reported in the literature.

4. References

1. Cui, B., Jia, S., Tokunaga, E., Shibata, N. (2018). Defluorosilylation of fluoroarenes and fluoroalkanes. *Nat. Commun.* 9, 4393. doi: 10.1038/s41467-018-06830-w
2. Zhou, J., Jiang, B., Fujihira, Y., Zhao, Z., Imai, T., Shibata, N. (2021). Catalyst-free carbosilylation of alkenes using silyl boronates and organic fluorides via selective C-F bond activation. *Nat. Commun.* 12, 3749. doi: 10.1038/s41467-021-24031-w
3. Zhang, J., Zhang, Y., Geng, S., Chen, S., Liu, Z., Zeng, X., He, Y., Feng, Z. (2020). C–O Bond silylation catalyzed by iron: a general method for the construction of Csp^2–Si bonds. *Org. Lett.* 22, 2669–2674. doi: 10.1021/acs.orglett.0c00633
4. Xu, Z., Chai, L., Liu, Z. Q. (2017). Free-radical-promoted site-selective C–H silylation of arenes by using hydrosilanes. *Org. Lett.* 19, 5573–5576. doi: 10.1021/acs.orglett.7b02717
5. Liu, X. W., Zarate, C., Martin, R. (2019). Base-mediated defluorosilylation of $C(sp^2)$−F and $C(sp^3)$−F bonds. *Angew. Chem. Int. Ed.* 58, 2064–2068. doi: 10.1002/anie.201813294
6. Yamanoi, Y., Nishihara, H. (2006). Rhodium-catalyzed silylation of *ortho*-functionalized aryl halides with hydrosilanes. *Tetrahedron Lett.* 47, 7157–7161. doi: 10.1016/j.tetlet.2006.08.001
7. Streitwieser, A., Xie, L., Wang, P., Bachrach, S. M. (1993). Carbon acidity. 77. Ion pair carbon acidities of some silanes in tetrahydrofuran. *J. Org. Chem.* 58, 1778–1784. doi: 10.1021/jo00059a031

5. Copies of NMR spectra

Biphenyl-4-yltriethylsilane (3aa)

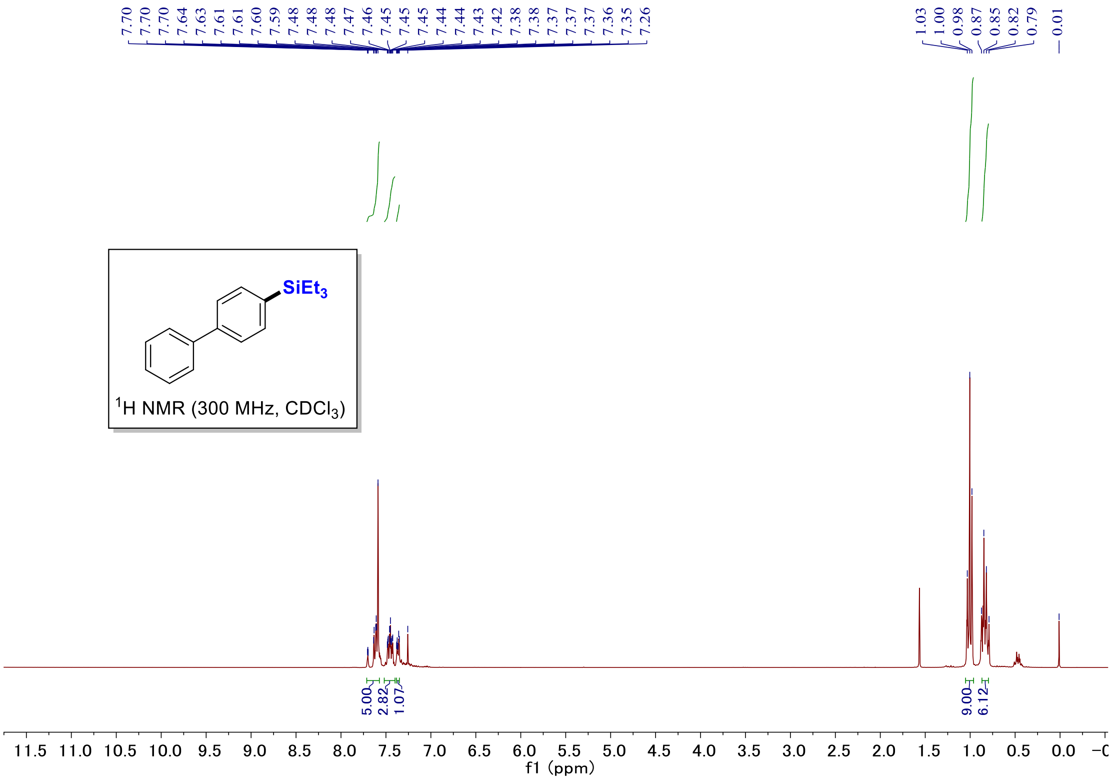

Biphenyl-3-yltriethylsilane (3ba)

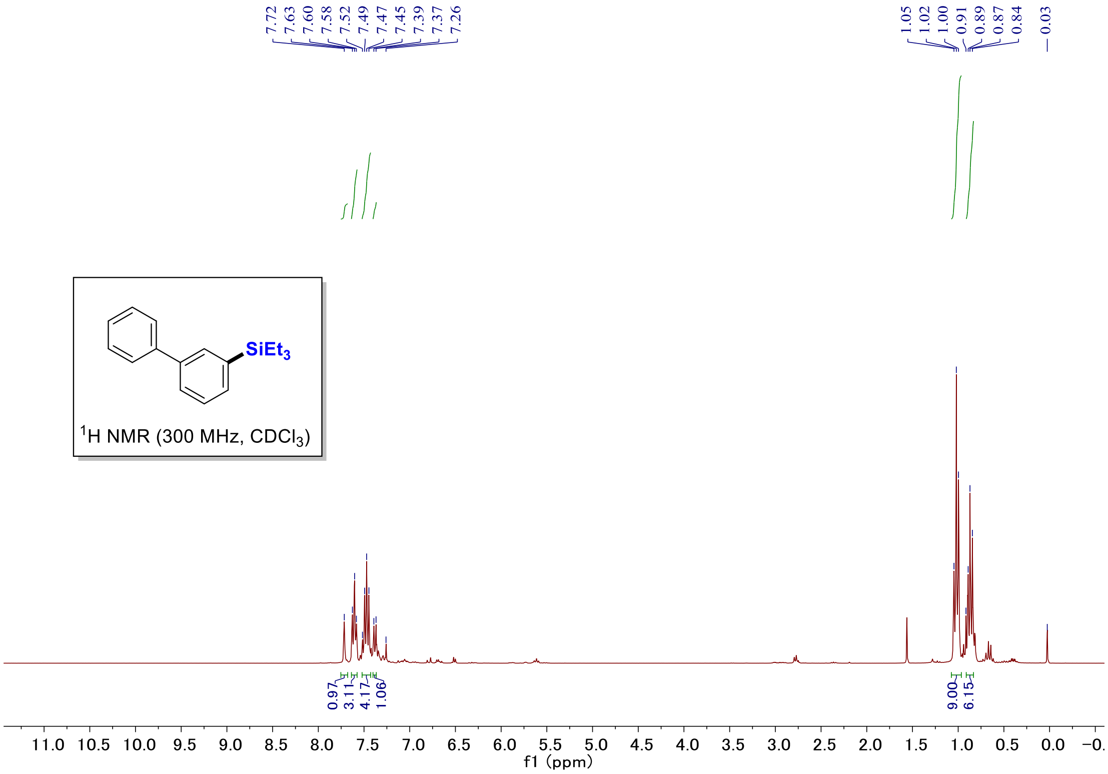

Biphenyl-2-yltriethylsilane (3ca)

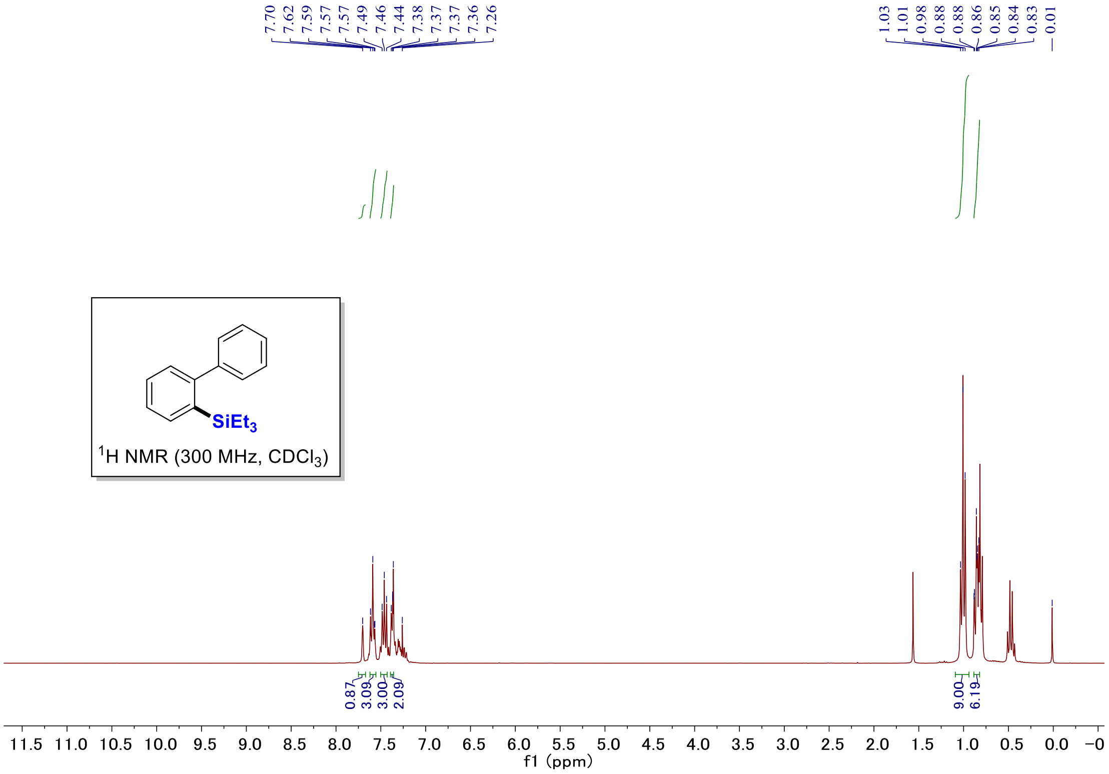

Triethyl(naphthalen-1-yl)silane (3da)

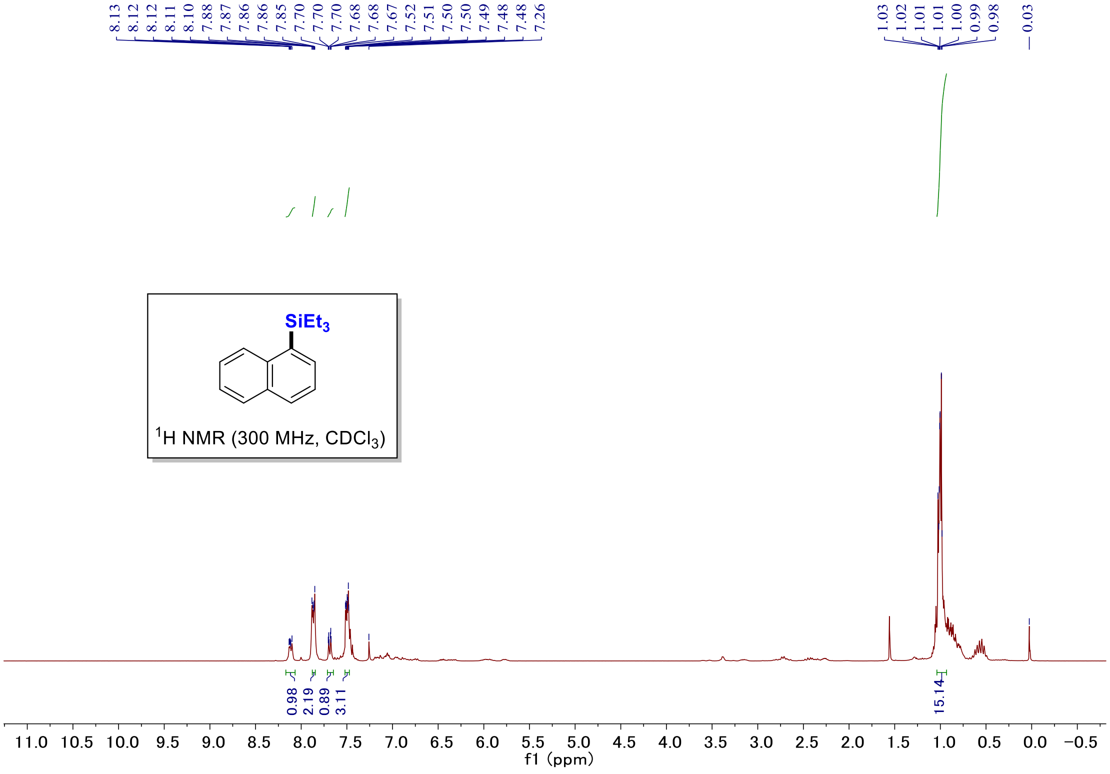

Triethyl(4-(naphthalen-1-yl)phenyl)silane (3ea)

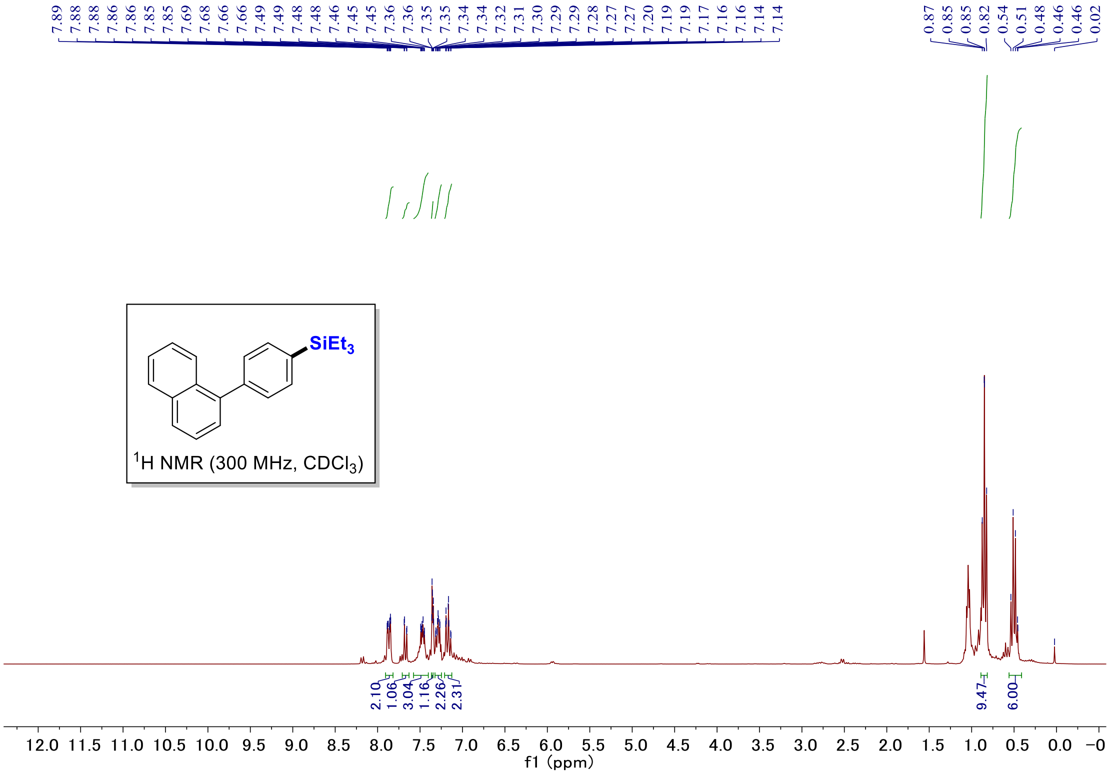

Triethyl(4'-methylbiphenyl-4-yl)silane (3fa)

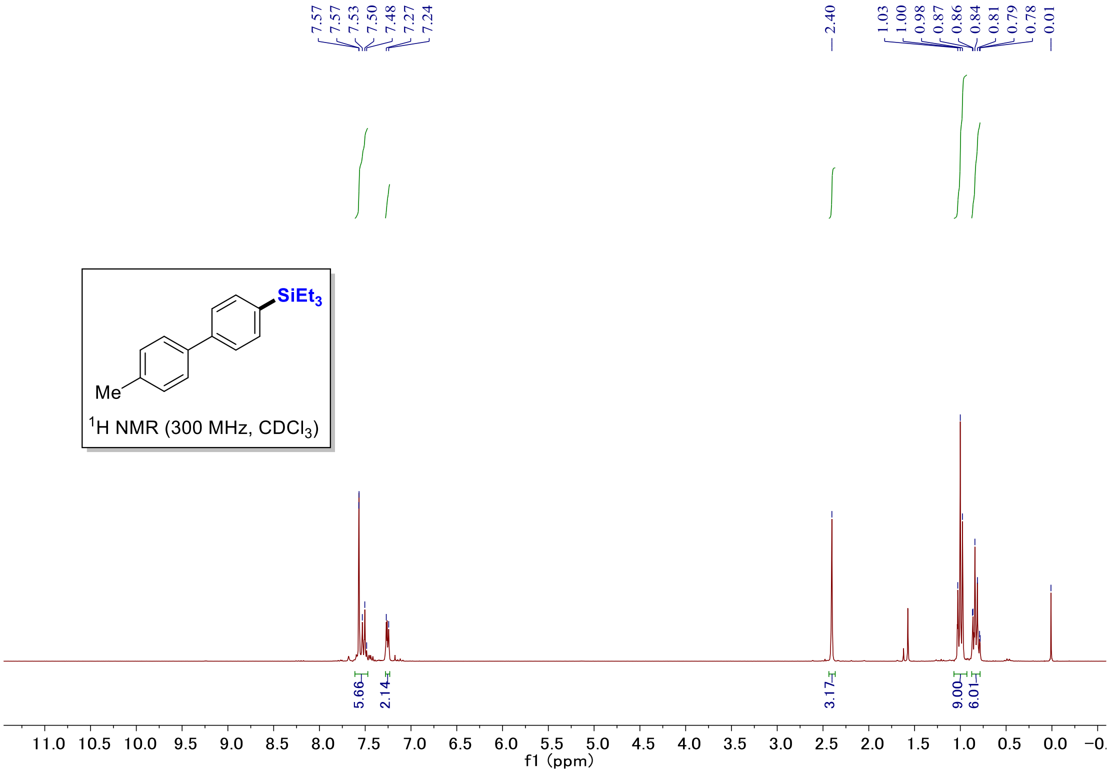

Triethyl(4'-methoxybiphenyl]-4-yl)silane (3ga)

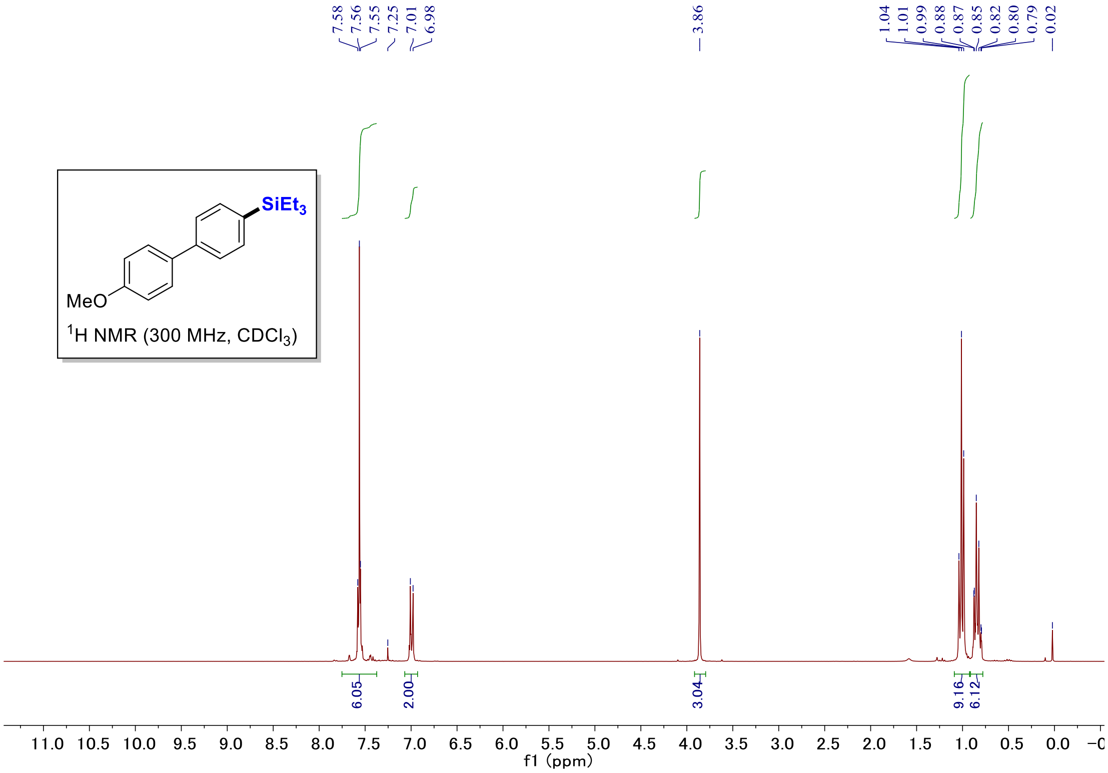

(4-(Benzo[*d*][1,3]dioxol-5-yl)phenyl)triethylsilane (3ha)

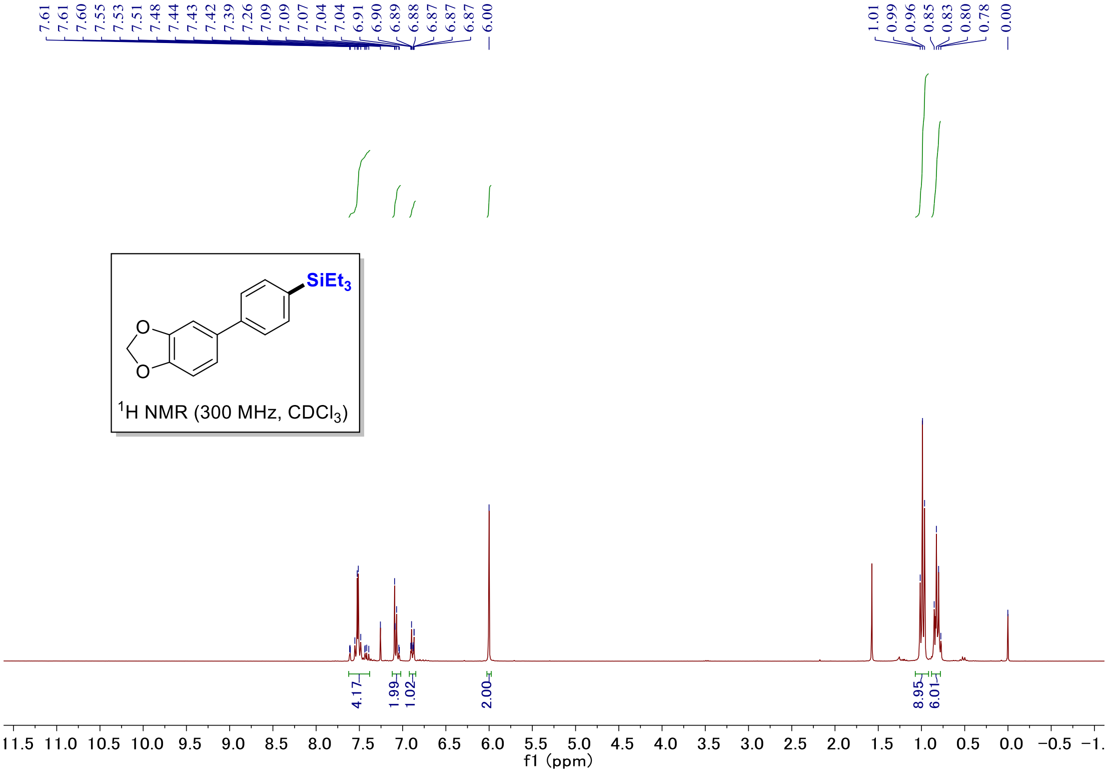

Triethyl(phenyl)silane (3ia)

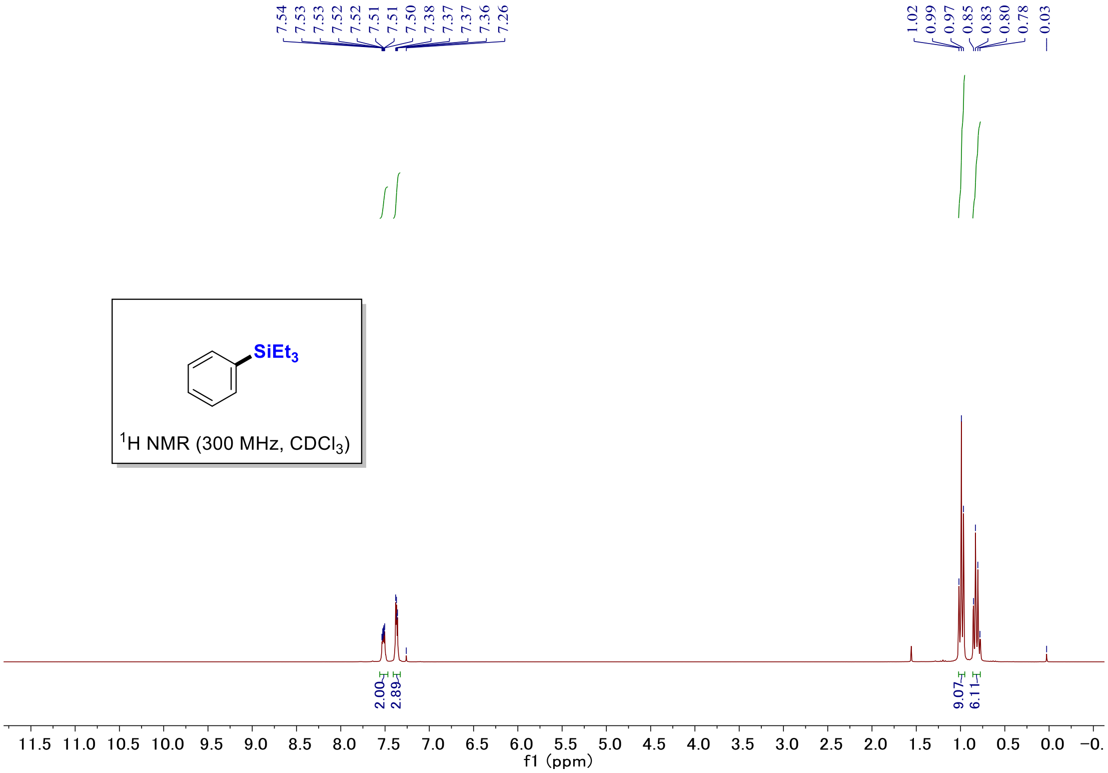

Triethyl(4-methoxyphenyl)silane (3ja)

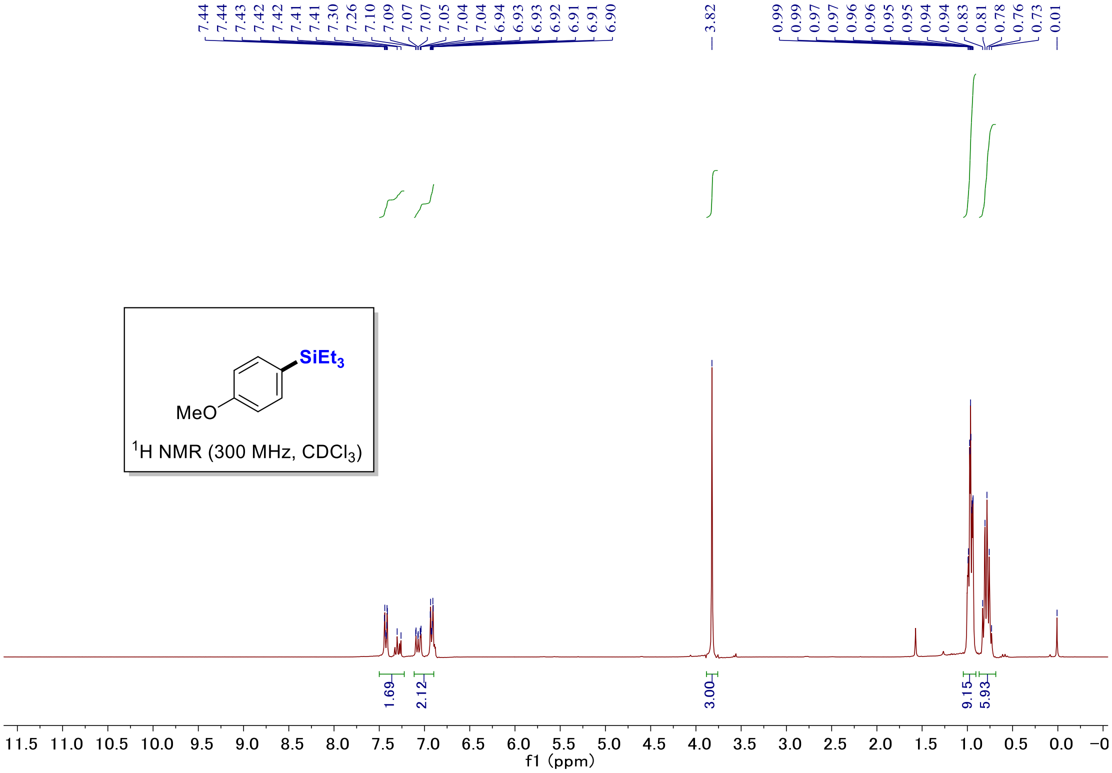

Triethyl(4-(methoxymethoxy)phenyl)silane (3ka)

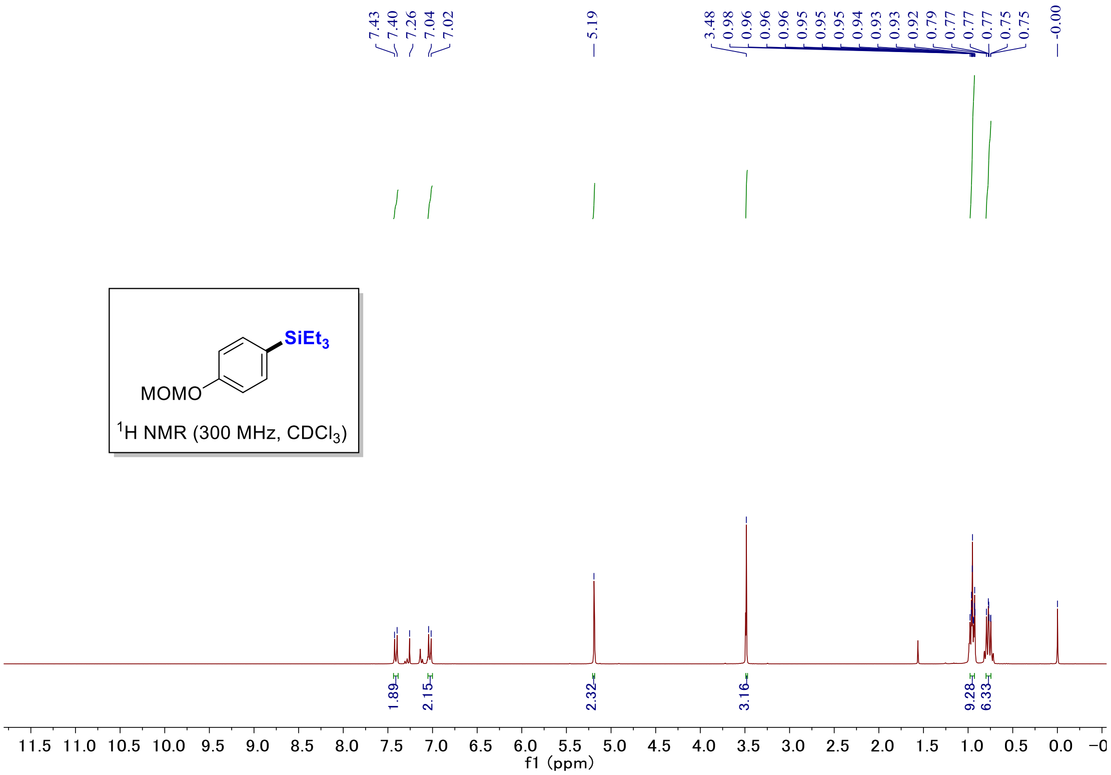

Triethyl(4-phenoxyphenyl)silane (3la)

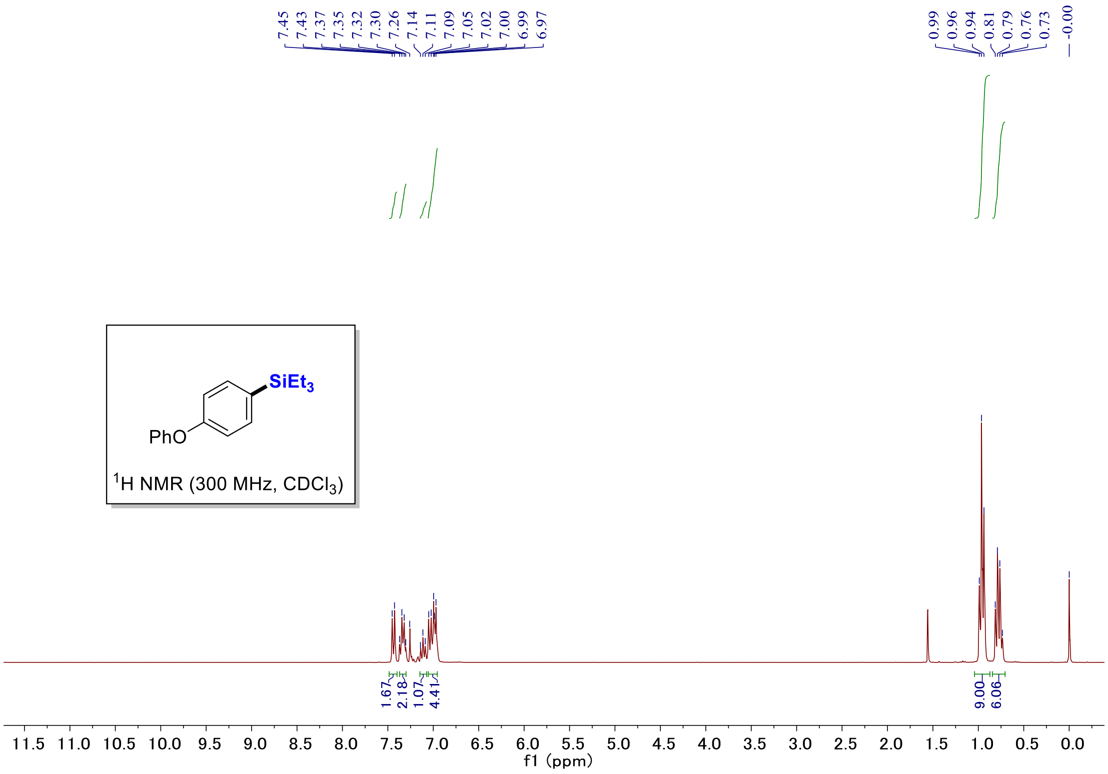

N,*N*-Dimethyl-4-(triethylsilyl)aniline (3ma)

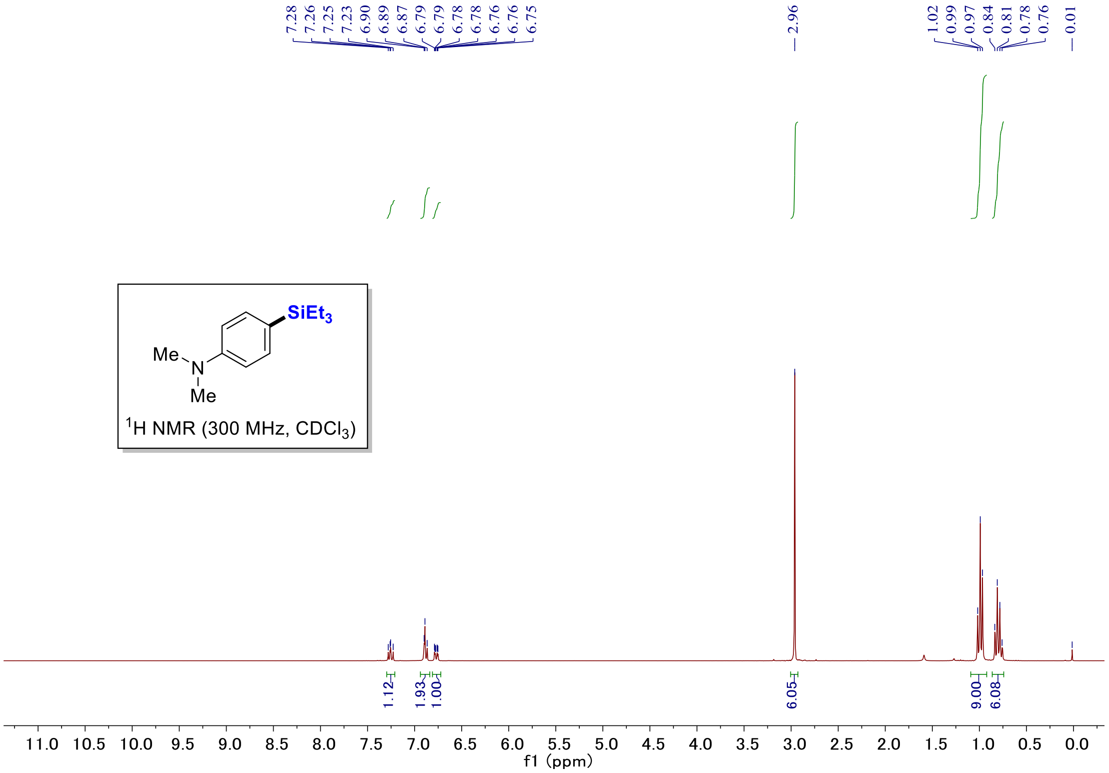

1-(4-(Triethylsilyl)phenyl)-1*H*-pyrrole (3na)

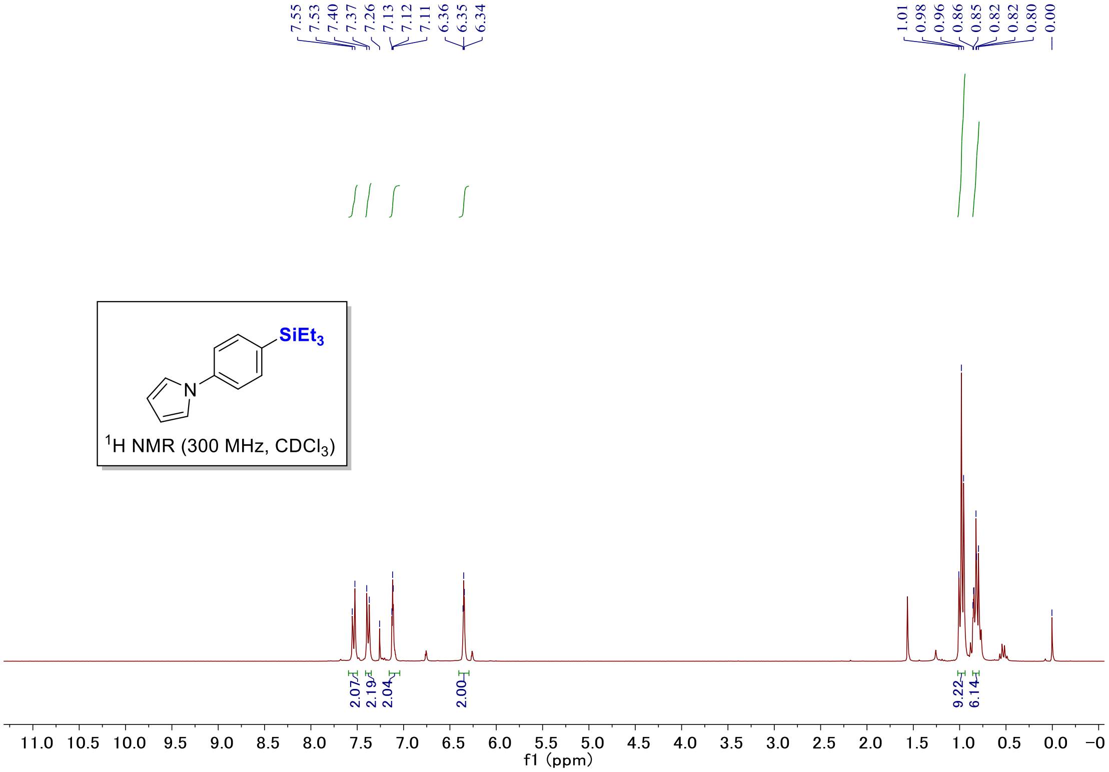

2-Phenyl-5-(triethylsilyl)pyridine (3oa)

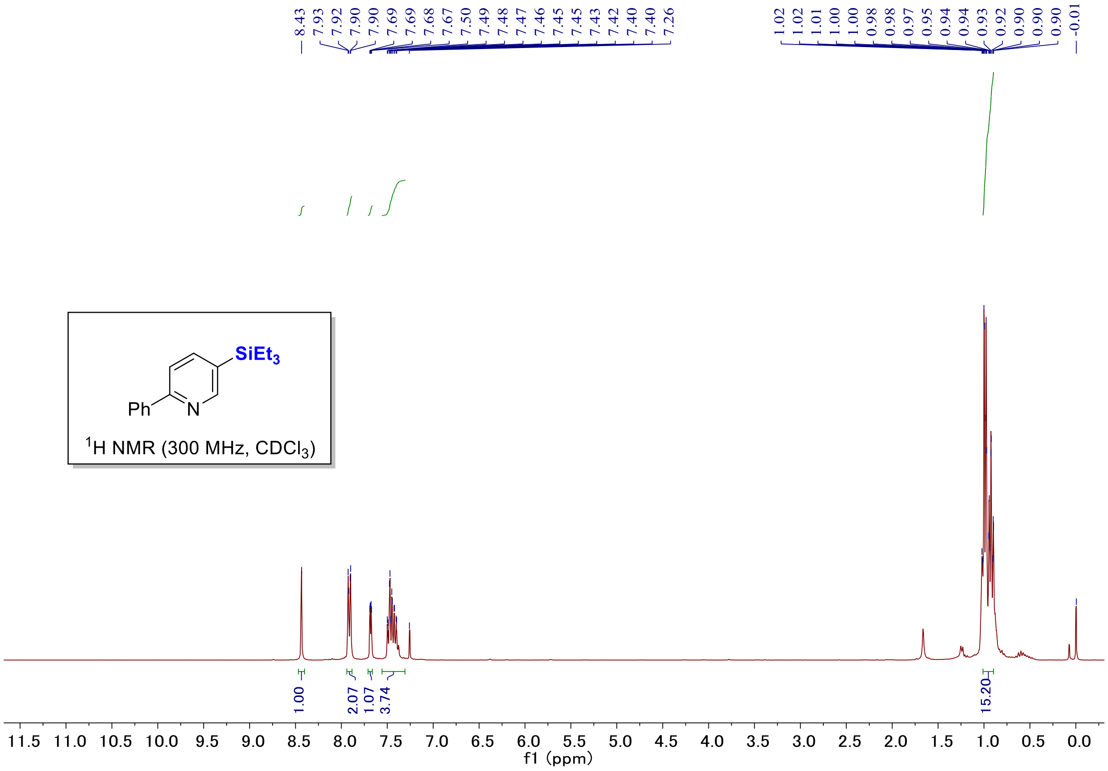

1-Methyl-6-(triethylsilyl)-1*H*-indole (3pa)

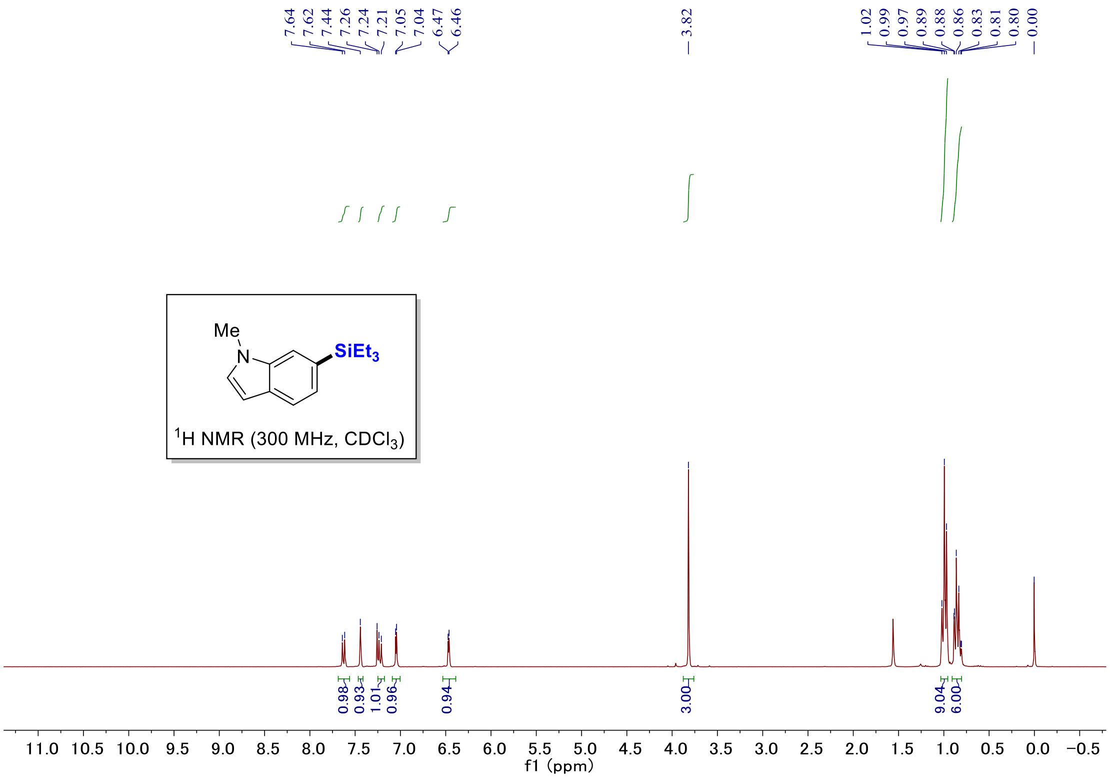

1-Methyl-2-(4-(triethylsilyl)phenyl)-1*H*-indole (3qa)

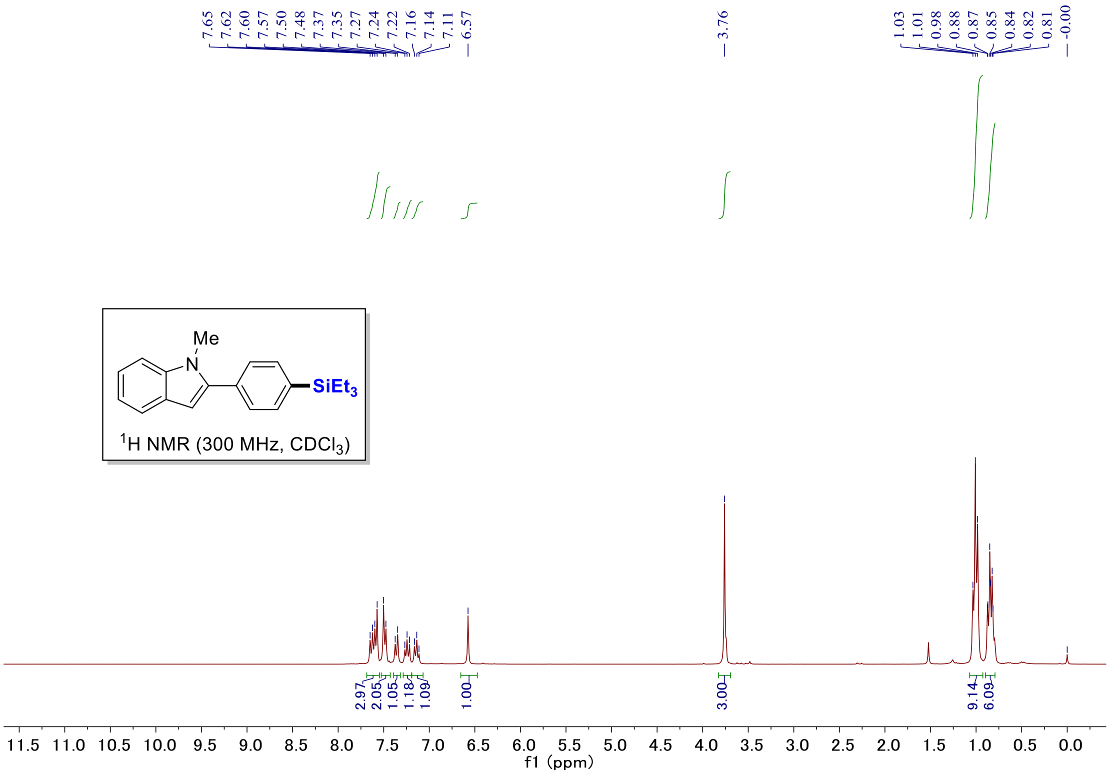

Triethyl(2-fluorophenyl)silane (3ra)

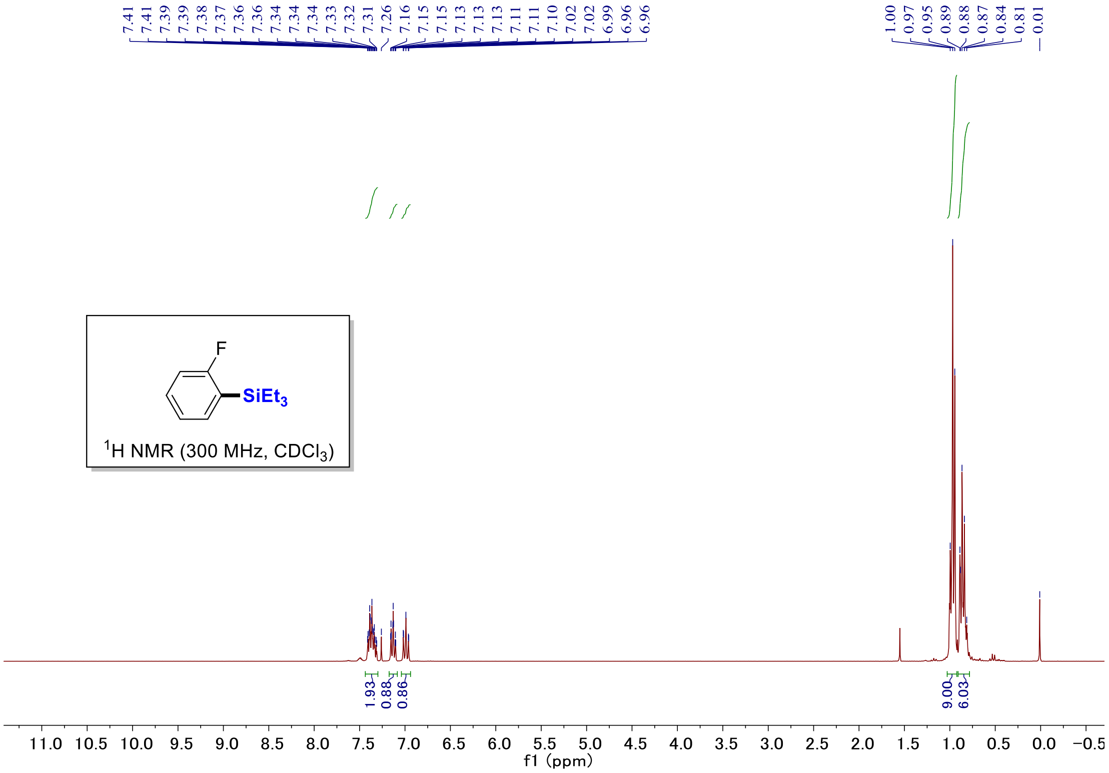

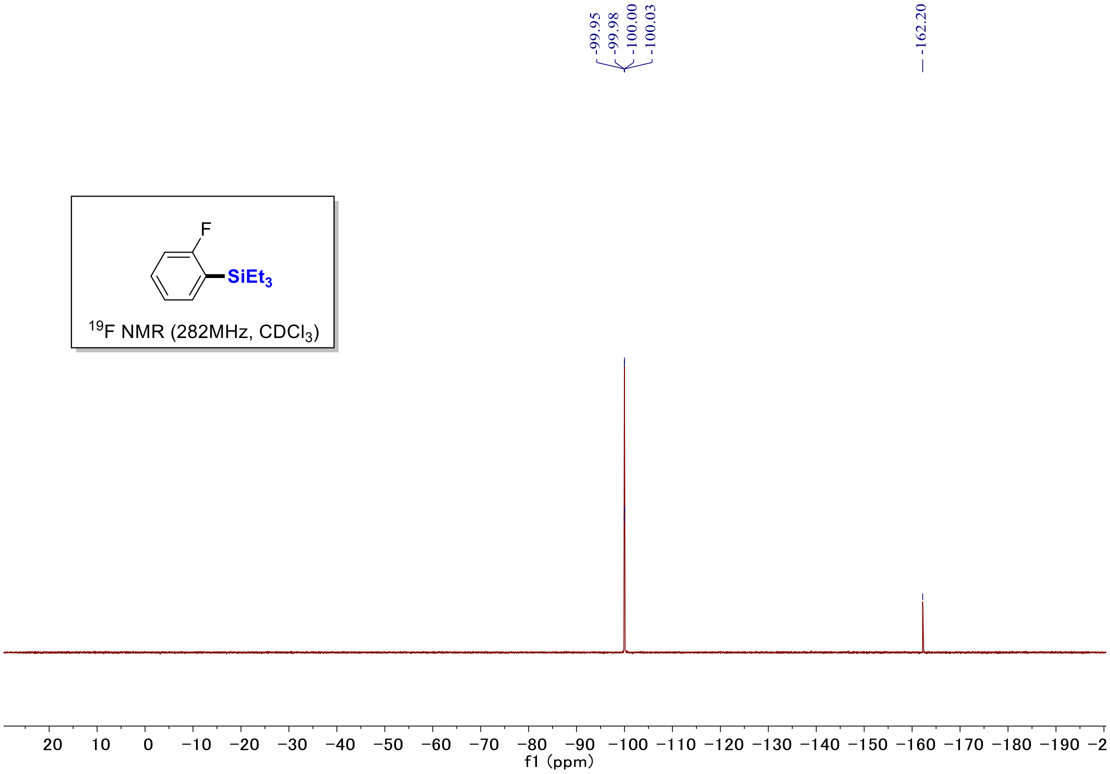

Triethyl(2-methoxyphenyl)silane (3sa)

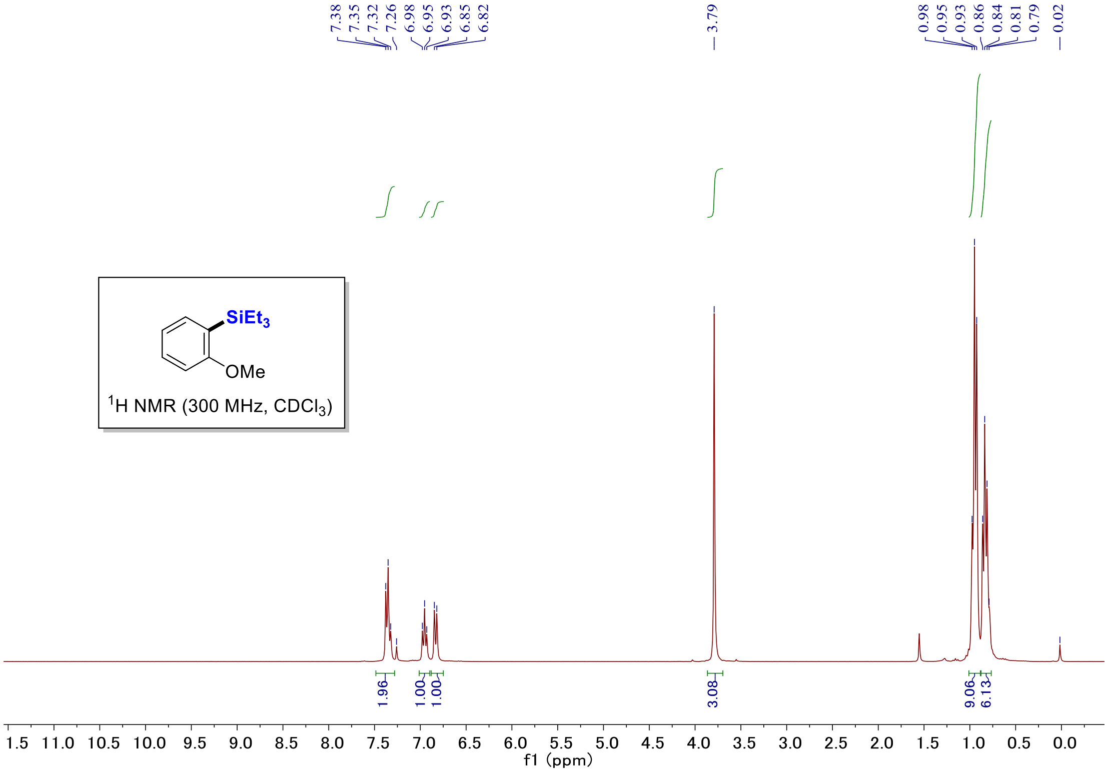

Triethyl(2-ethylphenyl)silane (3ta)

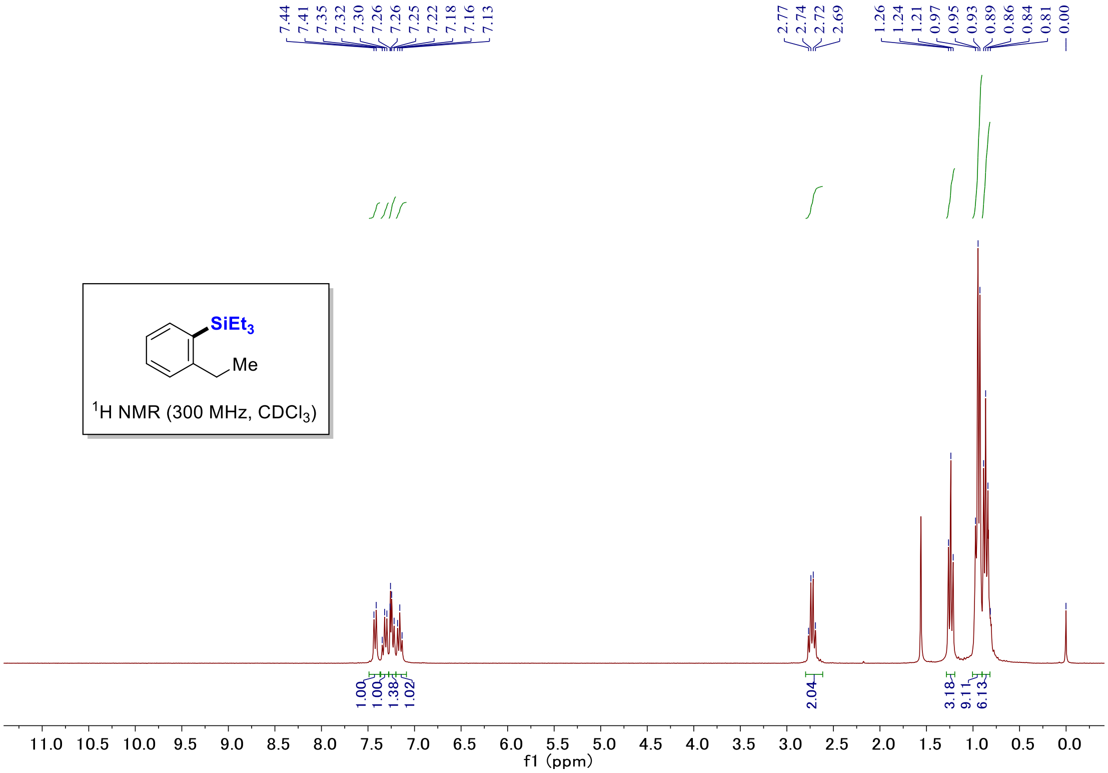

Biphenyl-4-yldimethyl(phenyl)silane (3ab)

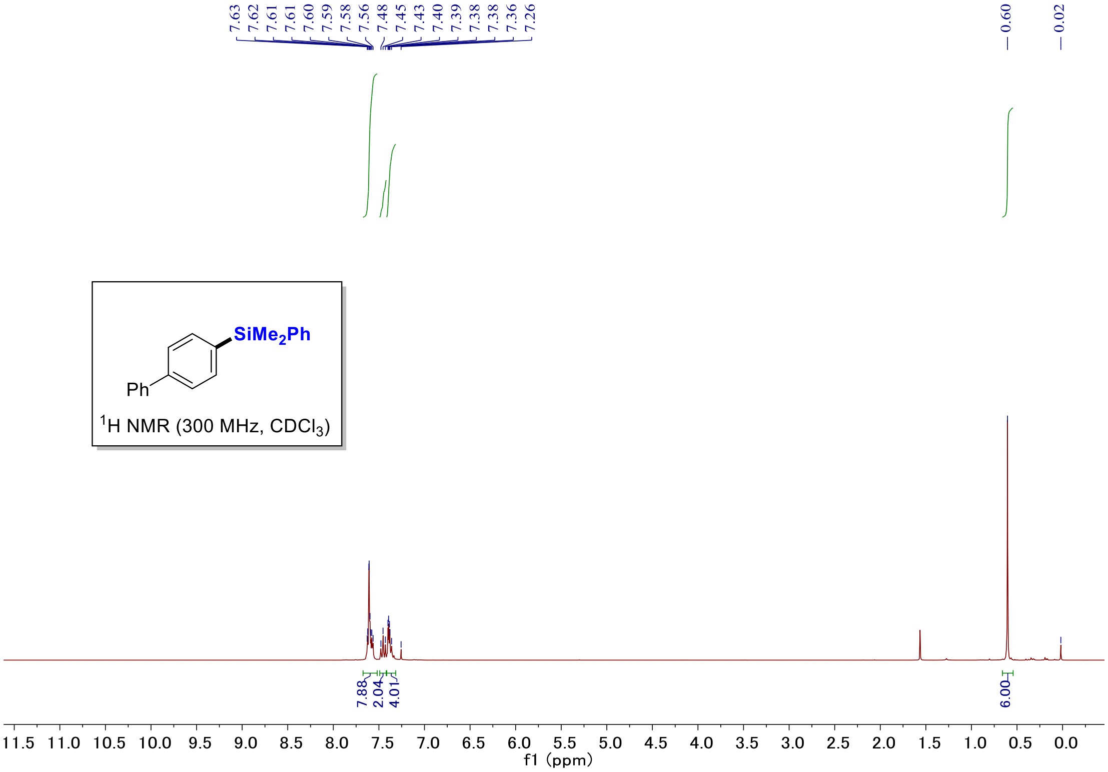

Biphenyl-4-yl(*tert*-butyl)dimethylsilane (3ac)

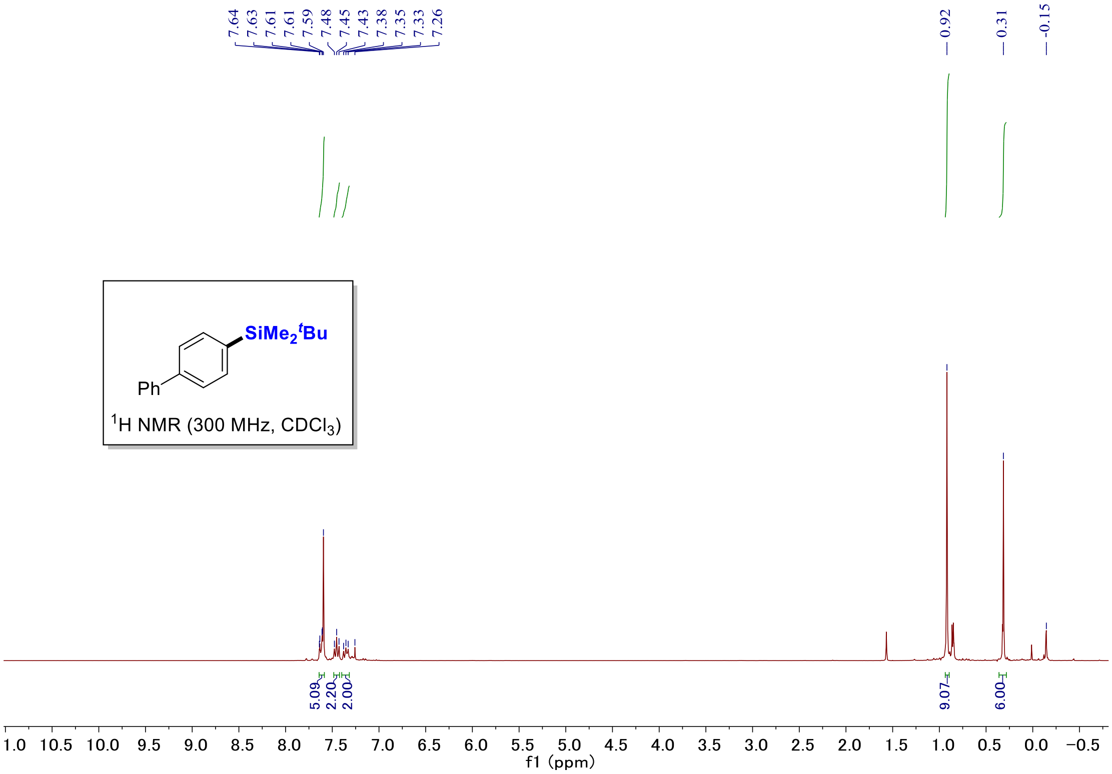

Supplement: Supplementary file 1 [file DataSheet1.PDF]
